# Molecular Signatures of Schizophrenia and Insights into Potential Biological Convergence

**DOI:** 10.3390/ijms26199830

**Published:** 2025-10-09

**Authors:** Malak Saada, Shani Stern

**Affiliations:** 1 Department of Neurobiology, University of Haifa, Haifa 3498838, Israel; malak.saada@gmail.com; 2 The Haifa Brain and Behavior Hub (HBBH), University of Haifa, Haifa 3498838, Israel

**Keywords:** schizophrenia, genetic architecture, epigenetic regulation, transcriptomic profiling, proteomic analysis, induced pluripotent stem cell (iPSC) models, biological pathways

## Abstract

Schizophrenia is a highly polygenic and clinically heterogeneous disorder. In this paper, we first review layer-specific evidence across genetics, epigenetics, transcriptomics, proteomics, and patient-derived induced pluripotent stem cell (iPSC) models, then integrate cross-layer findings. Genetics research identifies widespread risk architecture. Hundreds of loci from common, rare, and CNV analyses. Epigenetics reveals disease-associated DNA methylation and histone-mark changes. These occur at neuronally active enhancers and promoters, together with chromatin contacts that link non-coding risk to target genes. Transcriptomics show broad differential expression, isoform-level dysregulation, and disrupted co-expression modules. These alterations span synaptic signaling, mitochondrial bioenergetics, and immune programs. Proteomics demonstrates coordinated decreases in postsynaptic scaffold and mitochondrial respiratory-chain proteins in cortex, with complementary inflammatory signatures in serum/plasma. iPSC models recapitulate disease-relevant phenotypes: including fewer synaptic puncta and excitatory postsynaptic currents, electrophysiological immaturity, oxidative stress, and progenitor vulnerability. These same models show partial rescue under targeted perturbations. Integration across layers highlights convergent pathways repeatedly supported by ≥3 independent data types: synaptic signaling, immune/complement regulation, mitochondrial/energetic function, neurodevelopmental programs and cell-adhesion complexes. Within these axes, several cross-layer convergence genes/proteins (e.g., *DLG4*/PSD-95, *C4A*, *RELN*, *NRXN1/NLGN1*, OXPHOS subunits, *POU3F2*/BRN2, *PTN*) recur across cohorts and modalities. Framing results through cross-layer and shared-pathway convergence organizes heterogeneous evidence and prioritizes targets for mechanistic dissection, biomarker development, and translational follow-up.

## 1. Introduction

Schizophrenia is a complex neuropsychiatric disease with a multifactorial etiology including both genetic and environmental aspects [1,2,3]. Available data support the diathesis stress model as the primary causal theory, and genetic liability is strongly implicated [4,5]. New studies have investigated autoimmune and infectious mediators in some cases of schizophrenia [6]. Moreover, theories of neurodevelopmental and neurodegeneration processes [1], as well as lowered protein synthesis, have also been proposed [7]. Prenatal/perinatal adverse events, behavioral/neurocognitive signs during childhood, and schizotypal personality traits are correlated with an enhanced risk of developing schizophrenia [8]. Traditional diagnostic and treatment frameworks, largely reliant on clinical observation and trial-and-error pharmacotherapy, have proven insufficient for addressing the substantial interindividual variability in disease progression and treatment response [9,10].

Molecular psychiatry has made considerable progress over the past decades and is gradually increasing knowledge of the biological complexity of schizophrenia, allowing for the characterization of its genetic, epigenetic, transcriptomic, and proteomic landscapes. Genomic [11], epigenomic [12], and transcriptomic [13] analyses have also facilitated our understanding of disease risk profiles and molecular pathways [14,15]. Most risk is predicted by hundreds of common variants, and rare variants and copy number variants (CNVs) increased risk in a proportion of cases [11,16]. Concurrently, overlapping transcriptomic and epigenomic changes, particularly in synaptic plasticity, neuronal development, and immune signaling pathways, have also been observed in patient brain tissue and iPSC-derived neuronal models [17,18,19]. CNVs and new mutations are also involved in increased susceptibility to schizophrenia, especially among subpopulations. These include the well-characterized 22q11. 2 deletions and 16p11. 2 duplications, and they are also related to autism spectrum disorder and intellectual disability [20,21]. In schizophrenia research, proteomics remains less developed compared to other fields, with the identification of differential proteins and potential biomarkers having emerged only in recent years [22,23]. Integrating proteomic data with findings from other fields of study may provide a more comprehensive understanding of the disorder’s biological complexity [24,25].

iPSC models are emerging as a powerful tool for investigating the neurodevelopmental and molecular foundations of schizophrenia. A recent meta-analysis by Burrack et al. [26] identified consistent dysregulation of phosphodiesterase 4 (*PDE4*) gene expression in both brain and blood samples from individuals with schizophrenia. These findings were validated in iPSC-derived dentate gyrus-like neurons. This suggests a conserved molecular pathology across tissue types. In parallel, Stern et al. [27] demonstrated that monozygotic twins discordant for schizophrenia exhibit marked differences in neuronal maturation and synaptic transmission when modeled in iPSC-derived neurons. These findings support the role of early neurodevelopmental processes in disease emergence. Advancements in regional specificity have further enhanced the utility of iPSC models. For example, Sarkar et al. [28] developed a protocol for generating CA3 hippocampal neurons which enables in vitro modeling of hippocampal circuitry relevant to schizophrenia pathophysiology. This builds upon initial work by Brennand et al. [29], who first reported impaired connectivity and aberrant expression of genes involved in synaptic function and Wnt signaling in patient-derived iPSC neurons. Importantly, subsequent studies confirmed the translational validity of these models. Hoffman et al. [17] demonstrated that gene expression profiles in schizophrenia iPSC-derived neural progenitor cells (NPCs) and neurons recapitulate transcriptional signatures observed in post-mortem adult brain tissue. Beyond modeling schizophrenia in isolation, recent comparative studies have begun to uncover shared mechanisms with other related disorders. Romanovsky et al. [30] revealed significant genetic overlap between schizophrenia and autism spectrum disorder (ASD). In this study they showed that, although iPSC-derived neurons from these disorders follow distinct early developmental trajectories, they ultimately converge on similar synaptic impairments as they mature. Together, these findings highlight the value of iPSC models in capturing dynamic disease-relevant phenotypes.

The application of systems biology and machine learning has enabled integrative analysis, yielding testable hypotheses about disease mechanisms and candidate biomarkers for diagnosis and stratification [24,31]. These advances form the basis of a nascent but promising precision psychiatry paradigm; wherein molecular signatures could guide individualized treatment approaches. Notably, lymphoblastoid cell lines (LCLs) have demonstrated utility as predictive platforms in this domain. For instance, expression patterns of immunoglobulin genes in LCLs were shown to distinguish lithium responders from non-responders among individuals with bipolar disorder [32], while transcriptomic profiling of LCLs has been employed to predict suicide risk with promising accuracy [33]. More broadly, predictive models of drug response across a spectrum of psychiatric conditions have been proposed, highlighting the value of combining patient-derived cellular systems with genomic and transcriptomic data to refine treatment stratification strategies [34].

**Figure 1:**
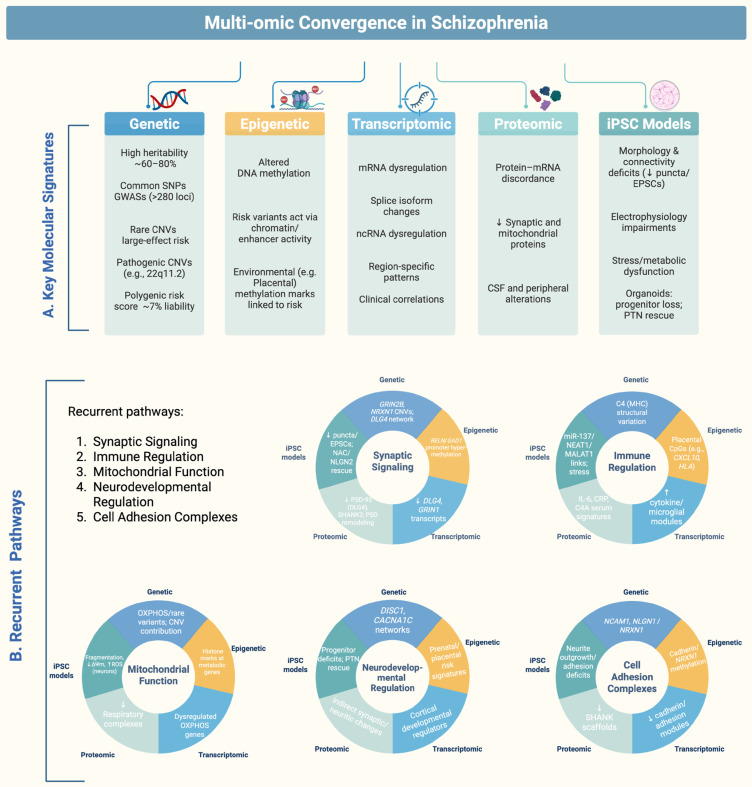
Multi-level molecular signatures implicated in schizophrenia and recurrent biological pathways. Panel (**A**): Key molecular signatures. Summary of convergent findings across five molecular layers. Genetic: high heritability and common/rare variant risk (GWAS, CNVs, rare coding). Representative sources: [3,16,35,36,37]. Epigenetic: altered DNA methylation and chromatin marks, including placenta-linked methylation associated with genetic risk: [12,38,39,40,41,42,43]. Transcriptomic: mRNA dysregulation, isoform/splicing changes, region/cell-type specificity (bulk and single-cell) [13,18,44,45]. Proteomic: protein–mRNA discordance with ↓ synaptic/mitochondrial proteins in cortex and alterations in peripheral fluids [46,47,48,49,50,51,52]. iPSC models: decreased synaptic puncta/EPSCs and electrophysiology changes; mitochondrial/oxidative stress; organoid progenitor loss with rescue (e.g., PTN) [29,53,54,55,56,57,58,59]. Panel (**B**): Recurrent molecular pathways. Evidence “wheels” indicate which layers support each pathway. (1) Synaptic signaling: transcripts/proteins and iPSC phenotypes (e.g., DLG4/GRIN1, ↓PSD-95/SHANK3; puncta/EPSC deficits) [13,29,47,48,55]. (2) Immune regulation: brain immune modules; peripheral cytokine/complement signatures (C4A) [38,49,50,60,61,62,63,64]. (3) Mitochondrial function: down-regulated OXPHOS transcripts, respiratory-complex protein changes, and iPSC mitochondrial phenotypes [44,46,58,65]. (4) Neurodevelopmental regulation: NPC/organoid disruptions with partial rescue by PTN/BRN2 [53,57,66]. (5) Cell-adhesion complexes: NRXN/NLGN/NCAM alterations across layers and models [29,53,54]. Abbreviations: ACC, anterior cingulate cortex; A1, primary auditory cortex; CNV, copy-number variant; CSF, cerebrospinal fluid; CRP, C-reactive protein; DMR, differentially methylated region; EPSC, excitatory postsynaptic current; GWAS, genome-wide association study; Hi-C, chromatin conformation capture; iPSC, induced pluripotent stem cell; IL-6, interleukin-6; lncRNA, long non-coding RNA; MHC, major histocompatibility complex; miRNA, microRNA; ncRNA, non-coding RNA; NPC, neural progenitor cell; OXPHOS, oxidative phosphorylation; PRS, polygenic risk score; PSD-95, postsynaptic density protein-95 (gene: *DLG4*); PTN, pleiotrophin; BRN2, POU class 3 homeobox 2 (gene: *POU3F2*); C4A, complement component 4A; SHANK3, SH3 and multiple ankyrin repeat domains 3. Conventions. Arrows (↑/↓) show direction vs. controls; gene symbols italicized; proteins in roman type. Colors match molecular layers across panels.

In this review, we examine schizophrenia’s molecular landscape across genetic and epigenetic variation, transcriptomic and proteomic alterations, and iPSC models. An overview of representative molecular alterations across these five domains is presented in Figure 1, which schematically summarizes key findings from genetic, epigenetic, transcriptomic, proteomic, and iPSC-based studies.

We define convergence as the recurring observation that heterogeneous upstream molecular alterations: genetic, epigenetic, transcriptomic, proteomic, or cellular, map onto a limited set of downstream biological pathways. We consider two complementary levels of convergence: (i) cross-layer convergence, where signals align within and between molecular layers, and (ii) pathway-level convergence, where diverse alterations aggregate onto shared biological pathways. These include synaptic signaling, immune regulation, mitochondrial bioenergetics, cell adhesion, and neurodevelopmental programs. Despite methodological and tissue-specific differences, these axes of dysfunction emerge repeatedly across levels of investigation, underscoring shared mechanisms of schizophrenia pathophysiology and the need for systematic validation through integrative, large-scale studies [13,16,18,19].

Recent reviews have offered comprehensive syntheses of specific molecular domains in schizophrenia, such as genetic architecture [67], epigenetic regulation [68], transcriptomic dysregulation [69], and iPSC-based modeling [70]. These have been invaluable in delineating each layer of biology but have generally considered them in isolation. This review synthesizes representative findings across individual molecular domains and then evaluates the broader concept of potential biological convergence. Evidence from synaptic, immune, mitochondrial, neurodevelopmental, and adhesion-related mechanisms recurs across multiple molecular layers, suggesting possible shared axes of vulnerability. While provisional, this perspective complements single-domain reviews and underscores patterns that merit systematic and quantitative validation in future research along with consideration of translational implications.

## 2. Genetic Risk Architecture of Schizophrenia

Schizophrenia is a strongly heritable neuropsychiatric disorder and is also polygenic [3]. Its heritability estimates derived from classical twin- and family-based studies are in the range of 60–80% [71]. Large-scale genome-wide association studies (GWASs) have enriched our knowledge regarding the genetic architecture of this complex disorder with the identification of >250 genome-wide significant loci. These loci are mapped to genes and regulatory elements implicated in pathways related to neurodevelopment, synaptic signaling, immune response, and calcium channel regulation, all of which are mechanisms implicated in schizophrenia pathophysiology [11,16]. Among these, variation within the major histocompatibility complex (MHC) region on chromosome 6 has received particular attention due to the functional characterization of the complement component 4A (*C4A*) gene. Structural variation at this locus affects *C4A* expression, which has been shown to correlate with schizophrenia risk and may contribute to altered synaptic pruning during adolescence [60].

### 2.1. Common Variation (SNPs and PRS)

Despite schizophrenia’s high estimated heritability (~80%) based on family and twin studies, isolating the specific genetic variants responsible for this risk has only became possible with large-scale GWASs. Since the first schizophrenia GWAS in 2008 [72] identified a common *RELN* variant increasing risk specifically in women, large-scale efforts have since expanded to identify over 100 genome-wide significant loci. Subsequent meta-analyses have increased this number to nearly 300 loci, many of which cluster in genes related to synaptic signaling, calcium channel activity, and immune modulation. For example, a landmark 2014 GWAS by the Psychiatric Genomics Consortium Schizophrenia Working Group, which included 36,989 cases and 113,075 controls, reported 108 significant loci, including genes such as *CACNA1C*, *MIR137*, and *GRIN2A* [11]. These findings were later extended in a meta-analysis involving over 320,000 individuals, identifying 287 loci enriched for pathways involved in synaptic signaling, calcium signaling, and immune regulation [16]. Most of the associated single nucleotide polymorphism (SNPs) are common variants with small individual effects on disease risk. However, their cumulative contribution is substantial, explaining approximately 23% of the SNP-based heritability of schizophrenia [11].

A polygenic risk score (PRS) is computed by summing an individual’s risk allele counts across thousands of common SNPs, each weighted by its GWAS-derived effect size, to yield a single metric of inherited liability to the trait [73]. In predominantly European-ancestry cohorts, the schizophrenia PRS accounted for ~7.7% of case–control variance corrected for 1% disease prevalence [11], much less than twin-based estimates of heritability (60–80%) [3,74]. People in the top decile of PRSs have a 3- to 4-fold odds of being diagnosed with schizophrenia relative to population expectation. PRSs have an AUC of nearly 0.65 in discrimination analyses [74]. That said, prediction accuracy is dramatically reduced in non-European populations, due to differences in allele frequency and linkage disequilibrium [75]. Novel multi-ancestry methods like PRS-CSx offer hope to enhance cross-population portability [76].

### 2.2. Rare Variation

Rare structural and coding variants beyond common risk variants contribute meaningfully to schizophrenia risk.

#### 2.2.1. Copy Number Variants (CNVs)

Recent large-scale genomic investigations have underscored the contribution of CNVs to schizophrenia risk. In a genome-wide analysis of over 21,000 schizophrenia cases and 20,000 controls, Marshall et al. [35] identified several recurrent CNVs significantly associated with disease susceptibility. The 22q11.2 deletion which is observed in approximately 0.3% of schizophrenia cases (OR: ~20–30×) encompasses key genes, including *TBX1*, which is implicated in neural crest development; *DGCR8*, which is involved in microRNA biogenesis; and *COMT*, critical for dopamine metabolism. This suggests that haploinsufficiency across this region may disrupt neurodevelopmental and neurotransmitter pathways. Similarly, 16p11.2 duplication, present in approximately 0.3% of cases (OR: ~9–10), affects genes such as *MAPK3* and *TAOK2*, which are components of the Ras/MAPK signaling cascade. In addition, it affects *KCTD13*, a regulator of RhoA signaling, with potential consequences for neuronal proliferation, synaptic plasticity, and cytoskeletal organization. These findings are consistent with prior reviews suggesting that rare, high-penetrance CNVs contribute to approximately 2–5% of the genetic liability for schizophrenia, primarily through perturbations of neurodevelopmental, synaptic, and circuit assembly pathways [20,21].

#### 2.2.2. Loss-of-Function Coding Mutation

Whole-exome sequencing (WES) of germline DNA from peripheral blood leukocytes of around 6000 schizophrenia cases and 6000 genetically matched controls revealed ultra-rare heterozygous protein truncating variants (PTVs) in genes encoding chromatin modifiers, transcription factors, and synaptic proteins [36]. Single-allele loss-of-function (LoF) mutations in *SETD1A* and *RBM12*, each with a prevalence less than 0.1%, demonstrate high penetrance (OR > 10×). This implicates mechanisms involving chromatin remodeling, histone modification, and gene-regulatory networks in schizophrenia pathogenesis [13,77].

A follow-up meta-analysis by the SCHEMA consortium confirmed these findings in 24,248 cases versus 97,322 controls. Among the identified genes were *GRIN2A*, *TRIO*, *CACNA1G*, and *SETD1A*, which are enriched for ultra-rare LoF variants conferring high risk. These genes are highly expressed in cortical neurons and are enriched in the following pathways: synapse assembly, glutamatergic signaling, and ion channel regulation [37]. Notably, *SETD1A* was identified in both studies, suggesting a consistent signal across independent cohorts and reinforcing its potential contribution to schizophrenia risk.

### 2.3. Gene Regulation and Functional Genomics

Functional genomic analyses have been key in understanding the molecular consequences of genetic risk. Fromer et al. [13] reported an RNA-seq and expression quantitative trait loci (eQTL) mapping analysis of dorsolateral prefrontal cortices (DLPFCs) from 467 post-mortem donors (n = 258 schizophrenia patients, 209 controls). They found that many schizophrenia risk variants are associated with gene expression levels in a range of brain areas which have been implicated in executive function and cognition, especially the DLPFC.

Similarly, Jaffe et al. [12] merged DNA methylation and transcription data with data for DLPFCs from 526 subjects, verifying that risk loci are enriched in non-coding enhancers active in neurons and glial cells. These regulatory changes largely localize to active enhancer marks (H3K27ac) in neuronal chromatin, emphasizing the impact of the levels of epigenetic and chromatin control. Furthermore, Pardiñas et al. [77] demonstrated that genes prioritized by GWAS and eQTL analyses are highly enriched for association with genes under strong evolutionary constraint, as defined by loss-of-function intolerance probability (pLI ≥ 0.9) in the ExAC reference of 60,706 exomes. This highlights the selective vulnerability of essential neurobiological processes to alteration.

Furthermore, Pardiñas et al. [77] demonstrated that genes prioritized by GWAS and eQTL analyses are highly enriched for association with genes under strong evolutionary constraint, highlighting the selective vulnerability of essential neurobiological processes to alteration.

### 2.4. Systems Biology and Pathway Convergence

Convergent signals across a range of genomic domains have emerged through integrative multi-omics analysis. Pathways and network analyses constantly identify synaptic plasticity; glutamatergic signaling; immune modulation, especially complement C4A signaling; and calcium channel activity as a part of the picture of the mechanism underlying the pathological processes of schizophrenia [11,16,60]. Variants in the MHC, including those controlling C4A levels, are particularly interesting because of a hypothesized involvement in exaggerated synaptic elimination during adolescence, which has been suggested to underlie the onset of schizophrenia symptoms during early adulthood [60]. Systems biology analyses further support the notion that genetically distinct individuals may converge on shared molecular networks, reflecting common downstream mechanisms despite heterogeneous upstream variation [24,78,79].

### 2.5. Genetic Subtypes and Network Modeling

Data-driven methods start to reveal genetically distinct schizophrenia subgroups that correlate with clinical features. For example, Arnedo and colleagues [80] analyzed three independent GWAS cohorts totaling 6500 cases and 8650 controls, using a kernel-based SNP-set test. This analysis identified five reproducible SNP sets, each mapping to biological pathways such as glutamatergic synaptic signaling and neurodevelopment. These genetic clusters were associated with differential symptom profiles, distinguishing subgroups predominantly characterized by positive versus negative or cognitive symptoms.

Another method for subgrouping patients was used by Birnbaum and Weinberger [31], who used pathway-specific polygenic risk clustering in a deeply phenotyped sample of 12,000 schizophrenia patients. By quantifying polygenic burden across synaptic function, immune signaling, and calcium channel pathways, they identified three distinct subtypes: “synaptic-loaded,” “immune-loaded,” and “mixed”. These labels reflect the dominant biological systems affected by the individual’s genetic risk burden, and the subgroups were found to differ in clinical features and antipsychotic treatment response rates, supporting their potential relevance to precision psychiatry. These emerging network-based and subtype discovery analyses suggest that molecular profiling can help characterize schizophrenia’s clinical heterogeneity and inform precision psychiatry interventions.

An overview of the major classes of genetic variants implicated in schizophrenia, their representative loci, frequencies, effect sizes, and related key biological pathways is provided in Table 1.

## 3. Epigenetic and Chromatin Regulation in Schizophrenia

Brain development and neuronal function heavily depend on epigenetic mechanisms, which include DNA methylation and histone modifications, as well as three-dimensional chromatin architecture, to regulate gene expression. Epigenetic mechanisms were shown to be involved in schizophrenia pathophysiology, and these mechanisms are essential disease contributors together with genetic variations [81,82]. Unlike genetic mutations, epigenetic modifications are dynamic and can be influenced by environmental exposures, making them a plausible link between external risk factors and long-term neurobiological changes. In schizophrenia, multiple human and model-system studies now converge on the idea that maladaptive epigenetic states both mediate genetic risk and translate environmental exposures into long-lasting molecular alterations [83].

### 3.1. Environmental Influences on the Epigenome

The fetal brain experiences long-term epigenetic changes because of environmental factors encountered during prenatal development. A growing body of work suggests that schizophrenia’s lifelong risk begins in utero, when maternal exposures leave lasting “marks” on the fetal epigenome [84,85]. The combination of maternal infection with malnutrition and psychosocial stress and hypoxia creates early neurodevelopmental problems which make individuals more prone to schizophrenia [85,86].

In a series of rodent experiments, Bale and colleagues [86] subjected pregnant mice to chronic psychosocial stress during the equivalent of the human first and second trimesters. They showed that offspring of stressed dams exhibited robust changes in DNA methylation and histone acetylation within hippocampal and prefrontal cortical neurons. These alterations persisted into adulthood and were accompanied by deficits in working memory and social interaction. These preclinical data provided evidence that early environmental insults can durably remodel the brain’s epigenetic landscape.

Supplementing animal studies, in a large meta-analysis of epidemiological studies of over a million births, Brown and Derkits [85] found that exposure to maternal influenza, nutritional deprivation, and hypoxia in utero were associated with offspring risk for schizophrenia, which was increased by 1.5–3-fold. These human data, derived from peripheral readouts, suggest that prenatal immune and metabolic stressors must perturb molecular mechanisms, likely epigenetically. These mechanisms generate stable alterations in developmentally programed neurodevelopmental trajectories.

Finally, Ursini and co-workers [38] provided integrative evidence for gene × environment interactions in schizophrenia by directly examining DNA methylation in the human placenta, a key fetal tissue mediating maternal influences. The study analyzed placental biopsies collected at birth from 82 individuals who were later diagnosed with schizophrenia and 75 matched controls. They reported enrichment of the immune-related (*CXCL10*, HLA loci) and oxidative stress-regulator (*NFE2L2*) methylation signatures at specific genes. In addition, they found a significant PRS-by-documented obstetric complications interaction effect for these same CpGs. Pathway analysis of these findings implicated dysregulated placental angiogenesis and cytokine signaling as G×E candidate nodes. These results suggest that epigenetic alterations in the placenta may mediate the biological effects of prenatal environmental stressors in the context of genetic risk. This suggests a mechanistic framework for the developmental origins of schizophrenia.

Together, these findings from animal models [86], population-scale epidemiology [85], and human placental epigenomic profiling [38] support a model in which prenatal exposures, including infection, nutritional deficiency, psychological stress, and hypoxia, may induce persistent epigenetic dysregulation in the fetal brain, thereby priming vulnerability to later psychopathology.

Beyond prenatal and placental influences, postnatal environmental stressors also play a significant role in shaping schizophrenia risk. Epidemiological and molecular studies have implicated urbanicity, cannabis use, and psychosocial trauma as contributors to disease vulnerability. These exposures are increasingly recognized as modulators of epigenetic states, influencing DNA methylation and stress-responsive transcriptional programs in both brain and peripheral tissues. Reviews by Roth et al. [82], Svrakic et al. [83], Migdalska-Richards and Mill [81], and Punzi et al. [87] emphasize that such postnatal exposures act as dynamic epigenetic modifiers, interacting with genetic liability to exacerbate molecular vulnerability. Considering both prenatal and postnatal exposures thus provides a fuller understanding of how environmental inputs converge on epigenetic dysregulation and contribute to schizophrenia pathophysiology.

### 3.2. Epigenetic Inhibitory Alterations in Post-Mortem Brain Tissue

Post-mortem studies of the prefrontal cortex have identified overlapping epigenetic dysregulation of GABAergic synaptic transmission in schizophrenia. In one of the first reports, Costa et al. [88] compared Brodmann area 9 (BA9) tissue from 14 individuals with schizophrenia and 14 non-psychiatric controls. They reported that the *RELN* promoter CpG island was approximately 50% more methylated in schizophrenia, accompanied by an ~50% reduction in *RELN* mRNA levels. At the same time, *DNMT1* transcript and protein levels were elevated by about threefold, implicating increased maintenance methylation as a mechanism for *RELN* repression.

Expanding on these observations, Gavin and Sharma [89] examined DLPFC (Brodmann area 46) samples (n = 15/group) donated by schizophrenia and control groups who were matched by race and other demographic characteristics. They revealed a 40% higher methylation of CpG islands on the *GAD1* promoter in schizophrenia, while chromatin immunoprecipitation identified a two- to threefold increase in *DNMT3A* enrichment at the same locus. These epigenetic modifications were associated with a nearly >35% reduction in *GAD1* (GAD67) transcriptions, linking de novo methylation to impaired inhibitory signaling.

More recently, Nishioka et al. [90] applied genome-wide MBD-capture sequencing to the DLPFC in 10 schizophrenia cases and 10 controls. They found widespread promoter hypermethylation in schizophrenia, particularly in *RELN*, *GAD1*, and other GABAergic genes, and identified by Western blotting that both DNMT1 and DNMT3A protein levels were almost twice as high in the same cohort.

Cumulatively, the described studies sampled nearly 40 schizophrenia and healthy control brains. Collectively, they indicate that upregulation of the maintenance methyltransferase *DNMT1* and/or the de novo enzyme *DNMT3A* within the PFC mediates hypermethylation and transcriptional silencing of key GABAergic genes (*RELN* and *GAD1*). Such changes are likely to contribute to reduced inhibitory signaling and the cortical circuit alterations observed in schizophrenia.

### 3.3. Histone Modifications and Regulatory Landscapes

Dysregulation of histone modifications besides DNA methylation in schizophrenia has also been proposed to affect the chromatin conformation at critical regulatory regions.

Among the earliest reports, Akbarian et al. [39] compared histone arginine methylation in post-mortem prefrontal cortices (Brodmann’s area 9) from 10 schizophrenia cases and 10 matched controls. They observed a roughly 2-fold increase in H3meR17 signals across promoters of metabolic genes, most notably glycolytic enzymes, accompanied by a 30–40% reduction in their mRNA levels. This raised the possibility that over-deposition of H3meR17 might suppress the neuronal metabolic pathways in schizophrenia and thereby lead to the disturbances of energy homeostasis in cortex circuits.

Building on these results, Jaffe et al. [12] performed ChIP-seq for the active enhancer mark H3K27ac inDLPFCs from 236 donors (n = 141 schizophrenia patients, 95 controls). They identified over 3000 DLPFC enhancers with significantly altered H3K27ac enrichment in schizophrenia. Differentially acetylated enhancers were strongly overrepresented near genes involved in synaptic plasticity (e.g., *BDNF* and *GRIN1*), cell adhesion, and immune signaling, including multiple MHC-linked loci.

More recently, Gusev et al. [40] combined H3K4me3 and H3K27ac ChIP-seq data for the prefrontal cortex (n = 50 schizophrenia patients, 50 controls) and showed that risk SNPs were enriched at enhancers marked by both H3K4me3 and H3K27ac in excitatory neurons. These “double-marked” regions are heavily enriched for genes mediating synaptic vesicle trafficking, actin cytoskeleton dynamics, and complement cascade-mediated pruning (including *C4A*), suggesting a mechanistic connection between genetic risk and disrupted chromatin states in pathways central to schizophrenia.

While many studies report significant changes in histone marks such as H3K27ac and H3K4me3 [12,40], others have failed to replicate these findings or observed them only in specific tissues or cohorts [39]. These inconsistencies highlight the need for larger and standardized ChIP-seq datasets to clarify the robustness of histone modifications in schizophrenia.

These ChIP-based studies, from small, focused analyses of histone arginine methylation to large-scale profiling of acetylation and trimethylation marks, further show that the pathological landscape of schizophrenia is characterized by changes in histone modifications at enhancers and promoters that regulate metabolism, synaptic stability, immune activation, and neurodevelopment.

### 3.4. Chromatin Architecture and Long-Range Interactions

In recent years, three-dimensional chromatin conformation studies have begun to reveal how non-coding schizophrenia risk variants perturb long-range regulatory interactions. Punzi et al. [87] applied promoter-capture Hi-C to post-mortem adult dorsolateral DLPFCs from six donors (three schizophrenia cases, three controls) and identified over 40,000 promoter–enhancer contacts in cis and trans. By intersecting these loops with 145 genome-wide significant schizophrenia loci, they showed that nearly 60% of risk haplotypes contact genes located hundreds of kilobases away, often skipping the nearest gene, and nominate new candidate effectors such as *GRIN2B* and *MEF2C* rather than the originally annotated loci.

Complementing this, Wang et al. [19] performed in situ Hi-C on homogenized adult prefrontal cortex tissue (five neurotypical donors). They detected ∼1.2 million chromatin loops, of which schizophrenia risk SNPs were significantly enriched at loop anchors (*p* < 1 × 10^−5^). For example, risk variants in an intergenic region on chromosome 12q13.2 formed a high-confidence loop to the promoter of *GRIN2B*, implicating glutamatergic signaling in disease etiology. Similarly, they reported a locus on 5q14.3 looped to *MEF2C*, a key regulator of synaptic plasticity and neuronal survival.

Additionally, Gusev et al. [40] integrated schizophrenia GWAS summary statistics with Hi-C data from iPSC-derived neural progenitors and cortical neurons (two lines per cell type) to perform “SNP2Gene” assignments via chromatin interactions. They found that roughly one-third of non-coding risk SNPs co-localize with physically linked gene promoters in neural cells, with a strong bias toward synaptic vesicle and chromatin-remodeling genes. This cell-type-specific loop mapping refines target gene prediction at known loci and highlights novel candidates such as *CACNA2D2* and *SYNGAP1*.

Together, these studies demonstrate that many schizophrenia-associated non-coding variants exert their effects by rewiring enhancer–promoter contacts in cortical neurons. This provides a direct mechanistic bridge from GWAS signals to dysregulated gene expression in pathways central to synaptic function and neurodevelopment.

### 3.5. Non-Coding RNAs and Peripheral Epigenetic Signatures

Several groups have now shown that schizophrenia is accompanied by consistent non-coding RNA and peripheral epigenetic signatures across both central and accessible tissues. In one of the first reports, Chen et al. [91] reviewed evidence from matched post-mortem DLPFC and blood-derived plasma studies in schizophrenia patients and controls. Across these primary investigations, miR-137 was consistently found to be reduced in both compartments, accompanied by de-repression of its targets, including genes involved in synaptic vesicle cycling (e.g., *SYN2*) and inflammatory signaling (e.g., *IL6R*). The same body of work reported upregulation of the long non-coding RNAs *NEAT1* and *MALAT1* in patient plasma, which correlated with elevated C-reactive protein and oxidative stress markers. Together, these findings suggest coordinated perturbations in miRNA–lncRNA networks that may contribute to schizophrenia pathophysiology.

Punzi et al. [87] extended these findings into a neurodevelopmental context by examining miRNA and lncRNA expression in cerebral organoids derived from iPSC models of five schizophrenia patients and five controls. They reported dysregulation of several miR-137 target networks, particularly those governing synaptic vesicle trafficking and innate immune signaling. They found that correcting miR-137 levels in organoids partially rescued neuronal arborization deficits. Alongside RNA regulators, locus-specific DNA methylation changes have emerged in more accessible tissues. Nishioka et al. [90] performed genome-wide methylation profiling of peripheral blood mononuclear cells (PBMCs) from 20 antipsychotic-naïve patients and 20 controls. They showed global hypomethylation and focal hypermethylation at the *DRD2* promoter. Importantly, the *DRD2* gene encodes the dopamine D2 receptor, a primary pharmacological target of antipsychotic drugs.

Taken together, these studies demonstrate that schizophrenia is accompanied by non-coding RNA and DNA methylation signatures in brain and peripheral tissues as well as patient-derived brain organoid models. These findings offer a promising, minimally invasive avenue for biomarker development despite ongoing challenges of tissue specificity and replication.

### 3.6. Therapeutic Potential of Epigenetic Modulation

Pharmacological targeting of chromatin regulators has emerged as a promising field in schizophrenia research. In an in vitro study on the effect of valproic acid (VPA) on chromatin, Gavin et al. [92] treated primary cortical neurons of Sprague–Dawley rat forebrains (n = 4) with VPA and revealed a 2–3-fold increase in H3K9ac and H3K14ac at the promoters of *BDNF* and *GAD1*. These findings correlated with an ~1.8-fold rise in their mRNA expression, demonstrating VPA’s capacity to promote a transcriptionally permissive chromatin state.

Extending these findings to human tissue, Guidotti et al. [93] examined post-mortem DLPFCs from schizophrenia patients (n = 12) who had received VPA and matched non-VPA-treated controls (n = 2). They found that VPA exposure was associated with enhanced H3K9ac enrichment at *RELN* and *GAD1* promoters and with increased expression of these GABAergic genes, which further supports the notion that VPA’s HDAC-inhibiting activity can reverse disease-related epigenetic repression.

In in vivo rodent models of stress and NMDA-antagonist-induced deficits, used as proxies for cognitive and social dysfunction, HDAC inhibition similarly yielded behavioral improvement. Covington et al. [94] subjected male C57BL/6J mice (n = 8 per group) to chronic social-defeat stress and then administered the class I HDAC inhibitor MS-275. Treated animals showed restoration of prefrontal synaptic proteins (PSD-95 and synapsin I) and normalized social-interaction behavior compared to vehicle controls. Likewise, Simonini et al. [95] gave adult Wistar rats (n = 10 per group) sodium butyrate for two weeks following MK-801-induced hyperlocomotion. Sodium butyrate reversed both the locomotor and Y-maze-assessed working memory deficits, illustrating that broad-spectrum HDAC inhibition can mitigate schizophrenia-relevant phenotypes.

Although broad-spectrum histone deacetylase (HDAC) inhibitors alter global chromatin, recent work has concentrated on more specific small molecules that directly affect DNA methylation or inhibit chromatin “reader” domains. Day and Sweatt (2000) treated rat organotypic hippocampal slices (n = 4) with the DNA methyltransferase inhibitor RG108 [96]. RG108 decreased methylation at the BDNF IV promoter by ∼40% and almost doubled BDNF mRNA expression without any cytotoxicity. Nestler et al. [97] subsequently treated trauma-exposed male C57BL/6J mice with the BET-bromodomain inhibitor JQ1 (n = 12/group) in the context of chronic unpredictable stress. JQ1 induced recovery from the stress-induced reduction in hippocampal synaptic proteins (GluA1 and PSD-95) and depressive-like behaviors in forced-swim and sucrose-preference assays, showing that specific inhibition of chromatin readers can particularly rescue synaptic resilience in vivo.

The combination of in vitro, ex vivo, and in vivo studies using rodent primary neurons, organotypic slices, cultured cells, post-mortem human brains, and live animal models demonstrates that pharmacological modification of histone acetylation, DNA methylation, and chromatin-reader proteins can reverse schizophrenia-related molecular and behavioral deficits. The preclinical evidence of efficacy in these agents supports the development of next-generation epigenetic psychopharmacology which uses targeted chromatin regulators to normalize dysregulated transcriptional programs and strengthen synaptic connectivity for improving cognitive and social function in schizophrenia patients.

An overview of the principal epigenetic and chromatin-based mechanisms implicated in schizophrenia, along with representative studies, tissue sources, regulatory impacts, and core pathways, is presented in Table 2.

## 4. Transcriptomic and RNA-Based Dysregulation

In addition to the genetic and epigenetic mechanisms discussed in Section 2 and Section 3, an emerging theme in schizophrenia is widespread abnormality of both coding and non-coding RNA species. Comprehensive RNA-seq profiling of major cortical and limbic brain regions has revealed strong, albeit regionally biased, alterations in expression that also implicate peripheral cells as a manifestation of systemic disease. For example, Collado-Torres et al. [44] analyzed post-mortem human brain tissue and reported approximately 245 and 48 differentially expressed genes (DEGs) in DLPFCs (n = 245 schizophrenia patients, 279 controls) and hippocampi (n = 48 per group), respectively, with little overlap between the two regions. Fromer et al. [13] examined the superior temporal gyrus in post-mortem samples (n = 40 per group) and reported aberrant synaptic-related transcripts, and Liu et al. [98] identified post-mortem amygdala DEGs (n = 22 schizophrenia patients, 24 controls) for immune and mitochondria-related processes.

### 4.1. Alternative Splicing

Aberrant alternative splicing and isoform expression are hallmarks of schizophrenia transcriptomics. Gandal et al. [18] performed RNA-seq on post-mortem frontal and temporal cortex samples (n = 258 schizophrenia patients, 301 controls) and identified over 3800 differentially expressed isoforms plus 515 splicing events, many within neurotransmitter signaling and immune-related genes. In a separate microarray study of DLPFCs (n = 100 schizophrenia patients, 100 controls), Wu et al. [99] reported more than 1000 differentially spliced genes and over 2000 promoter-usage shifts, including *DCLK1*, which encodes a microtubule-associated kinase involved in neuronal migration and axonal guidance during neurodevelopment and *PLP1*, a major component of central nervous system myelin, critical for oligodendrocyte differentiation and myelin sheath formation. Cohen et al. [100], using exon-junction arrays on post-mortem Brodmann area 10 samples (n = 40 schizophrenia patients, 40 controls), detected altered exon usage in the synaptic genes *ENAH* and *CPNE3*. Jaffe et al. [12], integrating post-mortem DLPFC RNA-seq (n = 191 schizophrenia patients, 335 controls) with genotype data, showed that many splicing changes co-localize with expression quantitative trait loci (eQTLs), indicating that schizophrenia risk variants can act via isoform-specific transcriptional mechanisms.

### 4.2. Non-Coding RNAs

Both miRNAs and lncRNAs are being increasingly reported as implicated in schizophrenia. In the first genome-wide small-RNA-seq study of post-mortem amygdalae (n = 13 schizophrenia patients, 14 controls), Liu et al. [98] observed global miRNA downregulation (notably, in the miR-1307 and miR-34 families). These changes were associated with de-repression of predicted synaptic and inflammatory targets. In another study conducted on LCLs (n = 20 schizophrenia patients, 20 controls), Sanders et al. [101] found an approximately 35% reduction in *DICER1* expression, implicating impaired miRNA biosynthesis as a potential mechanism. Mir-137 is a brain-enriched microRNA with demonstrated roles in neuronal development and synaptic plasticity. Olde Loohuis et al. [102] reported that miR-137 regulates neurodevelopmental processes and plasticity in rodent hippocampal neurons, identifying numerous downstream mRNA targets through gain- and loss-of-function experiments. Moreover, the MIR137 locus which encodes miR-137 is one of the most highly significant schizophrenia GWAS hits [103]. Gandal et al. [18] further identified lncRNA co-expression modules in DLPFC samples (n = 258 schizophrenia patients, 301 controls) enriched for immune and neurodevelopmental pathways. Importantly, Geaghan et al. [104] described sex-specific miRNA–mRNA interactions in PBMCs (n = 36 schizophrenia patients, 15 controls), highlighting additional layers of regulatory complexity.

Although downregulation of miR-137 and upregulation of lncRNAs such as *NEAT1* and *MALAT1* are among the most reproducible findings [87,91], other cohorts have reported null or inconsistent results, particularly for broader miRNA expression patterns [105,106]. Such variability underscores the influence of tissue type, sample size, and clinical heterogeneity and highlights the need for replication in larger and more diverse datasets.

### 4.3. Tissue Specificity and Peripheral Transcriptomic Profiles

Transcriptomic signatures in schizophrenia were often reported to be tissue-specific. For example, Collado-Torres et al. [44] analyzed post-mortem human brain tissue and demonstrated minimal overlap in DEGs across the DLPFC, hippocampus, Brodmann area 10, and superior temporal gyrus (n = 40–280 per region). In contrast, accessible peripheral cells such as LCLs (n = 20 schizophrenia patients, 20 controls) [101] and PBMCs (n = 36 schizophrenia patients, 15 controls) [104] exhibit reproducible immune-related expression changes. Although these peripheral profiles may reflect systemic or non-neuronal effects, they highlight possible opportunities for minimally invasive diagnostic biomarkers and longitudinal disease monitoring.

### 4.4. Co-Expression Networks and Systems-Level Convergence

Schizophrenia appears to involve coordinated dysregulation of gene modules, rather than isolated gene-level effects. In post-mortem DLPFC samples from 258 schizophrenia cases and 279 controls, Fromer et al. [13] applied weighted gene co-expression network analysis (WGCNA) to RNA-seq results. They identified large co-expression modules enriched for synaptic transmission genes. Importantly, these modules overlapped with genome-wide significant schizophrenia risk loci. Independently, Gandal et al. [18] reconstructed very similar modules in frontal and temporal cortices (258 cases, 301 controls) driven by both common and rare risk variants [77]. These modules have been proposed to act as intermediate molecular phenotypes, comprising synaptic, immune, and metabolic genes that may serve as convergence points for genetic risk, revealing how diffuse genetic liability converges on tightly co-regulated networks of synaptic, metabolic, and immune genes. Schizophrenia-associated modules have been reported to show reduced intramodular connectivity, meaning weaker co-regulation, in cases compared to controls, with similar patterns replicated in independent datasets [13,18].

A concise summary of key transcriptomic findings, sample types, affected genes, regulatory changes, and convergent pathways is provided in Table 3.

## 5. Proteomic and Functional Phenotypes

Building on the genetic, epigenetic, and transcriptomic landscapes outlined in Section 2, Section 3 and Section 4, proteomic analyses provide an additional layer of insight into schizophrenia by characterizing protein-level changes that may reflect downstream effects of molecular variation. Mass spectrometry studies of post-mortem cortical tissue have reported alterations in proteins associated with synaptic vesicle cycling, long-term potentiation machinery, and mitochondrial respiratory complexes [46,107]. Peripheral blood-based assays, including multiplex immunoassays, have identified immune-inflammatory profiles and candidate treatment-responsive protein panels. These findings suggest a possible correspondence between central proteome alterations and accessible biomarkers. In Section 5.1, Section 5.2 and Section 5.3, we synthesize these findings, spanning synaptic and mitochondrial proteome remodeling, immune and peripheral protein markers, post-translational modifications, and translational biomarker validation, highlighting potential points of convergence that could inform more personalized approaches to diagnosis and therapy.

### 5.1. Synaptic and Mitochondrial Proteome Disruption

Targeted proteomic analyses of postsynaptic density (PSD) fractions have been particularly informative for dissecting synaptic abnormalities in schizophrenia. Föcking et al. [47] applied shotgun proteomics to PSD isolates from the anterior cingulate cortex of 20 schizophrenia cases and 20 matched controls. They identified more than 700 PSD-associated proteins, of which 143 were differentially expressed, including key regulators of vesicle cycling and mitochondrial function, among them DNM1, MAPK3, and AP2B1, which are central to synaptic vesicle cycling, endocytosis, and long-term potentiation. Another study by MacDonald et al. [48] profiled the auditory cortex (n = 48 schizophrenia patients, 48 controls) and reported co-occurring differences in disturbances in PSD components (PSD-95 and SHANK3) and mitochondrial respiratory complexes. Together, these observations suggest that alterations in neurotransmission and cellular energy metabolism may occur in parallel in schizophrenia. Nevertheless, further studies are needed to clarify their extent and causal relationships.

### 5.2. Immune Signatures and Peripheral Protein Markers

Multiplex immunoassays have been widely applied to investigate peripheral protein alterations in schizophrenia, enabling the identification of candidate biomarkers with potential translational utility. Schwarz et al. [49] measured 181 proteins and small molecules by multiplex immunoassay in 250 first-episode or recent-onset schizophrenia patients, 280 healthy controls, and additional psychiatric groups (35 MDD, 32 bipolar, and 45 Asperger syndrome cases). In an initial discovery cohort of 71 schizophrenia versus 59 matched controls, they derived a 34-analyte “serum signature” comprising cytokines, growth factors, and endocrine markers. When tested across five independent cohorts, this panel classified schizophrenia cases versus controls with 60–75% accuracy and partially stratified MDD (~50%) and bipolar/Asperger cases (~10–20%). Ramsey et al. [108] assayed >100 analytes in serum from 150 schizophrenia patients (75 men, 75 women) and 150 controls, reporting 65 sex-specific protein differences in hormonal and inflammatory markers. Domenici et al. [50] conducted a plasma study of 229 schizophrenia patients and 254 controls, confirming that a panel enriched for immune- and metabolism-related proteins, notably IL-6, CRP, and BDNF, discriminated cases from controls. Beyond case–control stratification, proteomic profiling has also been studied to predict treatment response. Föcking et al. [62] showed, in a cohort of 30 amisulpride-treated patients, that treatment responders have elevated complement and coagulation factors (CFI, C4A, and VWF). This suggests that such profiles could potentially serve as peripheral indicators of treatment response. Elevated complement factors, particularly C4A, are of mechanistic interest because structural variation in the C4 locus has been genetically linked to schizophrenia risk. Increased C4A expression has been implicated in excessive synaptic pruning during neurodevelopment [60]. Collectively, these studies indicate that peripheral proteomic signatures encompass immune, hormonal, and metabolic alterations and that certain profiles may hold value both for diagnostic stratification and for guiding personalized treatment strategies.

Nonetheless, effect sizes for many peripheral biomarkers remain modest, and replication across independent cohorts has been variable. For instance, while panels enriched for IL-6, CRP, and related immune markers have discriminated cases from controls, their predictive accuracy typically falls short of clinical applicability, with classification accuracies in the range of 60–75% [49,50]. Similarly, complement factors such as C4A have been highlighted as potential treatment-response markers [47], yet their magnitude of change varies considerably between studies. Medication exposure represents an additional confounder, as antipsychotics can influence inflammatory and complement pathways, raising challenges for interpretation [109]. These limitations underscore the importance of larger, medication-naïve replication cohorts and standardized assay pipelines to validate immune and inflammatory proteomic signatures in schizophrenia [23,51,110].

### 5.3. Post-Translational and Signaling Modifications

Proteomic evidence also points to dysregulation of post-translational regulatory mechanisms in schizophrenia. Jaros et al. [52] performed phosphoproteomic analysis of plasma from 50 schizophrenia patients and 50 controls. The study revealed altered phosphorylation of acute-phase proteins and key intracellular signaling molecules, including Akt1 and STAT3. These changes may provide a mechanistic link between immune-inflammatory activation and downstream cellular signaling abnormalities. In a complimentary approach, Tomasik et al. [109] analyzed cerebrospinal fluid (CSF) from 20 cases and 20 controls and reported aberrant phosphorylation of coagulation factors and synaptic scaffolding proteins. Notably, several of these phosphorylation changes correlated with both symptom severity and antipsychotic exposure. These findings raise the possibility that altered post-translational regulation is associated with both disease processes and treatment effects.

Compared to genetics and transcriptomics, proteomics has progressed more slowly in schizophrenia research. This lag reflects technical challenges, such as the instability of proteins in post-mortem tissue, the lower sensitivity of earlier mass spectrometry platforms, and variability in sample processing. These issues contribute to modest effect sizes and limited reproducibility across cohorts. Recent innovations, however, are beginning to address these shortcomings. High-resolution mass spectrometry, single-cell proteomics, and spatially resolved proteomic approaches now offer the potential to detect cell-type-specific and regionally localized protein alterations. As these technologies mature, proteomics may begin to provide the same level of resolution and reproducibility as transcriptomics, thereby strengthening its role in defining convergent molecular pathways. Representative proteomic studies, analytical platforms, altered proteins, and implicated biological processes are summarized in Table 4.

## 6. Induced Pluripotent Stem Cell Models Elucidate Schizophrenia Pathophysiology

Patient-derived iPSC models now make it possible to interrogate schizophrenia’s cellular and molecular phenotypes in a human-specific context. Stern et al. [27] studied hippocampal neurons from monozygotic twins discordant for schizophrenia and reported that neurons from affected individuals had reduced arborization, hypoexcitability with immature spike features, and diminished synaptic activity. Neurons from unaffected co-twins displayed intermediate characteristics, reinforcing potential contributions from both genetic predisposition and post-zygotic factors. Similarly, earlier work by Brennand et al. [53] demonstrated that hiPSC-derived neurons from familial schizophrenia cases exhibit diminished neurite outgrowth, reduced synaptic marker expression, and impaired connectivity compared with controls. Subsequent work using a range of cellular models, including two-dimensional neuronal cultures, cerebral organoids, interneuron–glia co-cultures, oligodendrocyte precursor models, and astrocyte-neuron systems, has shown convergent disruptions in synaptic signaling, mitochondrial function, neurodevelopment, and glial support [54,55,56,57,58,66].

### 6.1. Early Neurodevelopmental Perturbations and Transcriptional Dysregulation

Neurodevelopmental abnormalities in schizophrenia can emerge as early as the neural progenitor stage. Brennand et al. [53] examined patient-derived forebrain neural progenitor cells (NPCs) from four schizophrenia and four control iPSC lines using both transcriptomic and quantitative proteomic approaches. They identified 312 differentially expressed genes and protein-level alterations linked to cytoskeletal remodeling and oxidative stress, including a 1.7-fold downregulation of *NCAM1*, a 1.5-fold downregulation of *NRXN1*, and a 1.6-fold downregulation of *NLGN1*, alongside a 2.2-fold increase in antioxidant-enzyme transcripts. Functionally, schizophrenia NPCs migrated 35% more slowly in neurosphere assays and produced 28% more reactive oxygen species in DCFDA assays. Complementing these findings, Ahmad et al. [66] reported altered microRNA expression in schizophrenia NPCs, with an ~1.8-fold elevation of miR-137 and an ~1.4-fold reduction in miR-9. This corresponded to reduced SOX2 and PAX6 protein levels and delayed MAP2 expression. The combined findings indicate that schizophrenia NPC phenotypes span coordinated transcriptional, epigenetic, and functional disruptions. These alterations at the NPC stage may compromise the timing and fidelity of early neuronal development.

### 6.2. Mitochondrial Malfunction and Oxidative Stress

Robicsek et al. [58] examined mitochondrial function in patient-derived neurons, reprogramming hair-follicle cells extracted from three schizophrenia and two control donors into dopaminergic (TH^+^) and glutamatergic (vGLUT1^+^) neurons. They observed a 30% increase in mitochondria fission, a 25% decrease in membrane potential, and a 35% increase in ROS. These changes were associated with a 20% reduction in neurite length. In extending these 2D neuron results, Kathuria et al. [56] probed mitochondrial function in 3D cerebral organoids from (n = 8 schizophrenia, 8 control) iPSC lines and showed a 22% decrease in basal O2 consumption rate and a 28% reduction in ATP-linked respiration, as well as a 40% lower Spike rate. While these studies differ in terms of model type, both point toward mitochondrial structural and functional alterations, along with oxidative stress, as potential cell-autonomous features in schizophrenia iPSC models.

### 6.3. Synaptic Connectivity and Dendritic Architecture

To assess synaptic structure and function, Brennand et al. [29] differentiated four schizophrenia (one 22q11.2del) and four control lines into cortical neurons. Immunostaining showed about 40% fewer PSD-95 puncta in patients’ neurons, and Sholl analysis revealed a 35% reduction in dendritic intersections. Whole-cell patch-clamp analysis recorded a 50% lower frequency of spontaneous EPSCs. Chronic treatment with loxapine increased PSD-95 density by 25% and EPSC frequency by 30%. In a parallel study, Pedrosa et al. [59] studied three schizophrenia vs. three control glutamatergic neurons and found sustained OCT4/NANOG expression up to day 60, with Synapsin-1 and Neurexin-1 cluster densities reduced by ~32% and ~28%, respectively, compared to controls. Extending these findings to a genetically controlled context, Stern et al. [27] examined hippocampal neurons derived from induced pluripotent stem cells of four pairs of monozygotic twins discordant for schizophrenia. Neurons from affected twins exhibited reduced dendritic complexity, fewer secondary and tertiary branches, and decreased total dendritic length. Electrophysiological recordings revealed lower spontaneous and evoked synaptic activity, reduced firing rates, diminished sodium and potassium current amplitudes, and immature action potential waveforms. Paired recordings and miniature EPSC analyses indicated both presynaptic and postsynaptic deficits, including reduced release probability and lower AMPA/NMDA current ratios. Notably, neurons from unaffected co-twins displayed intermediate values across parameters, suggesting that both shared genetic liability and that non-shared, potentially post-zygotic, influences may contribute to these cellular phenotypes. Collectively, these studies suggest that alterations in synaptic maturation and excitability are recurring features in patient-derived neuronal models of schizophrenia.

### 6.4. Organoid and Interneuron Circuit Deficits

Investigating network-level vulnerabilities, Kathuria et al. [55] co-cultured cortical interneurons from nine schizophrenia and nine control lines with excitatory neurons. Schizophrenia interneurons showed a 30% reduction in VGAT^+^ puncta, 42% lower GAD67, 38% lower gephyrin, and 45% lower NLGN2 by immunoblotting; either NLGN2 overexpression or N-acetylcysteine restored synaptic puncta and increased the mean firing rate by ~50%. Building on this circuit-level framework, Notaras et al. [57] profiled 25 organoids from nine schizophrenia and five control donors. TMT proteomics identified 150 proteins altered >1.5-fold, including a 50% drop in BRN2 and a 60% drop in PTN in schizophrenia organoids. Adding pleiotrophin for 7 days increased BRN2 levels by 2.2-fold, improved progenitor survival by 40%, and doubled NeuN^+^ neuron counts. Together, these studies point to network-level vulnerabilities in both 2D interneuron co-cultures and 3D organoids. This indicates that certain synaptic and progenitor-survival deficits in schizophrenia models can be partially ameliorated by targeted molecular interventions.

### 6.5. Oligodendrocyte Precursor Cell Dysfunction

Oligodendrocyte precursor cells (OPCs) are immature glial cells capable of differentiating into myelinating oligodendrocytes, thereby playing a critical role in central nervous system development, axonal insulation, and white matter repair [111]. While most patient-derived iPSC studies in schizophrenia have focused on neuronal phenotypes (see Section 6.2, Section 6.3 and Section 6.4), glial impairments have also been reported. In a family-based investigation, de Vrij et al. [54] generated NG2^+^ oligodendrocyte precursor cells (OPCs) from three CSPG4-mutation carriers with schizophrenia and three unaffected siblings to probe glial contribution to disease pathology. Patient-derived OPCs accumulated a threefold increase in high-mannose NG2 species and mislocalized NG2 within the Golgi, but the core myelin proteins MBP and PLP1 were reduced by 45% and 50%, respectively. The expression levels of key transcriptional regulators of OPC maturation (*SOX10* and *OLIG2*) were each ~30% lower in schizophrenia-derived OPCs. These findings suggest that OPC and myelin-gene dysregulation may occur alongside the synaptic, neurodevelopmental, and mitochondrial alterations described in earlier subsections, which suggests that multiple cell types could contribute to schizophrenia-related pathology.

At the molecular level, iPSC-based studies pinpoint specific genes and pathways that are repeatedly implicated across some schizophrenia models. These include alterations in synaptic assemblies, energy metabolism, cell-adhesion complexes, and myelin-gene networks. Such findings, while still limited in number, suggest recurring biological themes that merit further validation. Patient-derived iPSC models now enable interrogation of schizophrenia’s cellular phenotypes in a human-specific context. In Section 7, we integrate these cellular and molecular insights to explore potential avenues toward precision medicine in schizophrenia.

Despite their promise, iPSC-derived models face important limitations. Line-to-line variability introduces challenges in reproducibility, while the developmental immaturity of neurons and organoids limits their capacity to fully recapitulate adult brain circuitry. Moreover, current systems lack environmental inputs such as immune, hormonal, and psychosocial stressors, which are central to schizophrenia risk. Cerebral organoids and co-culture models extend the toolbox by capturing aspects of cellular diversity and synaptic connectivity, yet they too are constrained by variability in differentiation protocols, absence of vascularization, and limited maturation. A more systematic evaluation of these limitations will be critical for interpreting findings and for advancing next-generation patient-derived models.

An overview of iPSC-based models, cell types, observed phenotypes, and their relevance to schizophrenia pathology is presented in Table 5.

## 7. Systems Integration: Convergent Molecular Pathways and Cross-Layer Convergence Genes in Schizophrenia 

Findings from genome-wide studies, epigenomic profiling, transcriptomics, proteomics, and patient-derived cellular models indicate that, despite methodological and tissue-specific differences, recurrent biological themes emerge. We integrate evidence at two complementary levels: (i) a pathway-level matrix aggregating signals across genetics, epigenetics, transcriptomics, proteomics, and iPSC models (Table 6; see also Figure 1B), and (ii) molecule-focused case studies (Section 7.6) where evidence triangulates across ≥3 distinct molecular layers. Our goal is a synthesis of recurring signals, not a formal meta-analytic test.

### 7.1. Synaptic Signaling

GWASs have repeatedly highlighted synaptic genes like *CACNA1C*, *GRIN2A*, and *DLG2*, each contributing modest risk [11,77]. Rare copy number variants (e.g., 22q11.2 deletions) and high-penetrance loss-of-function alleles (*SETD1A*) further implicate synaptic assembly and plasticity networks [20,37]. In the epigenome, neuron-specific H3K27ac and H3K4me3 landscapes are enriched for schizophrenia risk variants at enhancers of postsynaptic genes [41], while DNA methylation shifts in the DLPFC affect synaptic loci [12]. Transcriptome-wide association studies (TWAS) link synaptic gene expression changes to schizophrenia risk loci [40], and Hi-C maps connect these loci to *GRIN2B* and *MEF2C* promoters [42]. At the transcript level, co-expression modules enriched for synaptic transmission and receptor scaffolds are disrupted in DLPFC [13,18], with isoform-specific splicing of *DLG2* and *SHANK2* further skewing synaptic composition [18]. Proteomic surveys confirm depletion of PSD-95, SHANK3, and other postsynaptic density proteins in anterior cingulate and auditory cortices [47,48], and phosphoproteomic studies in patient serum report altered phosphorylation patterns implicating dysregulated kinase signaling [52]. Finally, patient-derived iPSC neurons recapitulate these deficits: cortical cultures show ~40% fewer PSD-95 puncta, 50% fewer spontaneous excitatory currents, and partial rescue with loxapine [29], while interneuron co-cultures highlight NLGN2-dependent GABAergic synaptic failures [55].

### 7.2. Mitochondrial Bioenergetics

Genetic evidence links mitochondrial function to schizophrenia. De Vrij et al. [54] identified rare variants in mitochondrial electron transport chain (ETC) genes in familial schizophrenia cases. Notaras et al. [57] reported that patient-derived cerebral organoids display downregulation of oxidative phosphorylation (OXPHOS) genes, particularly in neurons and astrocytes, and show increased vulnerability to metabolic stress. Epigenetically, Akbarian et al. [39] examined post-mortem PFC tissue from schizophrenia patients and found reduced histone H3 arginine 17 dimethylation (H3R17me2) at promoters of genes encoding metabolic enzymes, alongside reduced transcription of mitochondrial and glycolytic pathway genes. At the transcriptomic level, Collado-Torres et al. [44] analyzed DLPFC samples from 258 schizophrenia cases and 279 controls, revealing coordinated downregulation of OXPHOS co-expression modules, implicating respiratory complexes I–V. Proteomic studies indicate that mitochondrial respiratory complex subunits are co-regulated with synaptic proteins and reduced in schizophrenia cortex. Föcking et al. [47] reported decreased abundance of NDUFV2 (complex I) and UQCRC1 (complex III) in the anterior cingulate cortex of schizophrenia patients. MacDonald et al. [48] similarly observed reduced ATP5A1 (complex V) in the primary auditory cortex, which correlated with reduced PSD-95 levels [47,48]. Functional modeling confirms mitochondrial deficits. In vitro, iPSC patient-derived dopaminergic and glutamatergic neurons exhibit a 30% increase in mitochondrial fragmentation, a 25% membrane potential decrease, and a 35% reactive oxygen species increase [58]. Three-dimensional cerebral organoids have a 22% decreased basal oxygen consumption rate and a 40% decrease in spontaneous neuronal spiking frequency relative to controls [56].

### 7.3. Cell-Adhesion Complexes

Genetic evidence implicates both common SNPs in *NCAM1*, *NRXN1*, and *NLGN1* and rare CNVs in cell-adhesion pathways critical for neurite outgrowth and synapse formation [11,53]. Epigenetic analyses demonstrate hypermethylation and transcriptional silencing of *RELN* in the prefrontal cortex, potentially disrupting extracellular matrix interactions and synaptic organization [88,89]. Transcriptomic profiling reveals widespread dysregulation of protocadherins and adhesion factor isoforms across cortical regions in schizophrenia [18]. Proteomic investigations identify altered abundance and phosphorylation of adhesion scaffolds, including AP2B1 and DNM1, in both serum and brain tissue from affected individuals [47,52]. Functional modeling in induced iPSC systems shows that forebrain neural progenitor cells (NPCs) derived from schizophrenia patients migrate approximately 35% more slowly than in controls and exhibit 1.5–1.7-fold lower expression of *NCAM1*, *NRXN1*, and *NLGN1* [53]. Oligodendrocyte precursor cells (OPCs) from CSPG4-mutation carriers display NG2 mislocalization alongside reduced expression of myelin proteins MBP and PLP1 [54].

### 7.4. Immune Regulation

Variants in the major histocompatibility complex (MHC), particularly complex structural variation in the *C4A* gene, increase complement activity and are strongly associated with excessive synaptic pruning in schizophrenia [60]. The human placenta and peripheral blood have exhibited immune-gene methylation signatures that interact with polygenic risk and obstetric complications, suggesting a combined genetic and environmental contribution to immune dysregulation [38,90]. Transcriptomics in both the brain and periphery, including large-scale RNA-seq analyses of post-mortem cortices and exon-array profiling DLPFCs, highlight upregulated cytokine and microglial modules in schizophrenia [18,101]. Co-expression network analyses integrating thousands of brain transcriptomes position immune genes, particularly those in complement and interferon pathways, at the core of schizophrenia risk modules [19,43]. Serum proteomic assays identify reproducible “immune signatures” (IL-6, CFI, and C4A) that stratify cases and forecast treatment response [49,62]. Patient-derived iPSC organoids and interneuron co-cultures similarly show dysregulated complement and cytokine pathways. These alterations were shown to be partially normalized by pleiotrophin supplementation or N-acetylcysteine treatment [55,57].

### 7.5. Neurodevelopmental Regulation

Schizophrenia risk is evident during prenatal and early postnatal brain development, where both inherited variants and environmentally induced epigenetic alterations converge to disrupt neuronal differentiation and progenitor viability. Recurrent 22q11.2 microdeletions and rare loss-of-function variants in *POU3F2* (*BRN2*) and pleiotrophin (*PTN*) implicate transcriptional regulators that govern cortical patterning and cell–matrix interactions [54,57]. Genome-wide methylation profiling of the human placenta identified differentially methylated regions at *PAX6* and *SOX2*. This demonstrates that obstetric complications interact with genetic liability to modify developmental histone marks and DNA methylation landscapes [38]. In vitro, induced pluripotent stem cell-derived neural progenitor cells from schizophrenia patients exhibit reduced expression of *NCAM1*, *NRXN1*, and NLGN1; dysregulated miR-137/PAX6 signaling; delayed neuronal marker expression; impaired migration; and elevated oxidative stress [53,66]. Three-dimensional cerebral organoids recapitulate these phenotypes, displaying approximately 50% reductions in BRN2 and PTN protein levels; importantly, exogenous pleiotrophin supplementation or BRN2 overexpression restores progenitor survival and increases neuronal differentiation [57]. Collectively, these data define a neurodevelopmental axis in schizophrenia whereby genetic perturbations and prenatal epigenetic modifications intersect to derail early cortical maturation.

### 7.6. Cross-Layer Convergence Genes/Proteins

Several genes emerge repeatedly across genetic, epigenetic, transcriptomic, proteomic, and patient-derived cellular studies, indicating that they may occupy central nodes within core pathways of schizophrenia.

- DLG4 (PSD-95):

Although not itself a GWAS hit, the PSD-95 scaffold shows concordant dysregulation at multiple levels: reduced H3K27ac at its enhancer in the DLPFC [12]; decreased mRNA in the DLPFC [13]; lower protein abundance in the anterior cingulate and auditory cortex [47,48]; and fewer PSD-95 puncta accompanied by reduced spontaneous EPSCs in patient-derived cortical neurons, which are rescued by loxapine [29].

- C4A/C4B:

Complement components encoded in the MHC region stand out across modalities: complex structural variation at C4A/C4B associates with risk [60]; enhancer marks H3K4me3/H3K27ac at C4 loci are enriched in schizophrenia neurons [40]; elevated C4A transcript levels appear in immune-gene co-expression modules [18]; and higher serum C4A/C4B protein distinguishes treatment responders [62].

- NRXN1/NLGN1:

These presynaptic adhesion molecules are disrupted genetically by rare CNVs and GWAS signals [11,20]; their mRNA is downregulated in patient NPCs [53]; and their puncta/clusters are reduced in iPSC-derived cortical and glutamatergic neurons [29,59].

- MT-CO1 and ATP5A1 (OXPHOS subunits):

Key components of mitochondrial respiratory chain complexes I–V show downregulation at the protein level in post-mortem cortices [47], and fragmented low-potential mitochondria with increased reactive oxygen species are present in patient iPSC neurons [58].

- RELN:

The reelin adhesion cue is epigenetically silenced by promoter hypermethylation in BA9 (14 vs. 14) [88], shows reduced mRNA in the DLPFC [89], and is reactivated at both the chromatin and transcriptional levels in VPA-treated patients [93].

- BRN2 (POU3F2) and PTN (pleiotrophin):

These two factors involved in neuronal differentiation and adhesion are consistently downregulated in schizophrenia organoids [57]; exogenous pleiotrophin or BRN2 overexpression rescues progenitor survival and neuronal output in these 3D models [57].

These case studies summarize recurring cross-layer signals linked to synaptic scaffolding, immune–synaptic interfaces, adhesion, mitochondrial function, and developmental regulation. They motivate prospective, quantitative tests to define effect sizes, boundaries, and translational utility (Table 7).

## 8. Discussion

Schizophrenia’s molecular biology has been profiled across genetics, epigenetics, transcriptomics, proteomics, and patient-derived iPSC models. Read together, these data repeatedly implicate five axes supported by multiple layers: synaptic signaling, immune/complement biology, mitochondrial/energetic function, neurodevelopmental programs, and cell-adhesion complexes. We use “convergence” in a conservative, hypothesis-generating sense: a cross-layer synthesis of recurring signals that nominates testable mechanisms (Figure 1B; Section 7).

**Figure 2:**
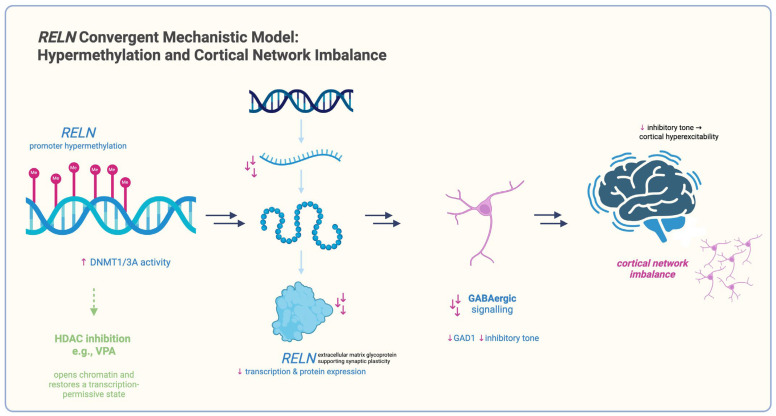
*RELN* convergent mechanistic model in schizophrenia. The above figure is a schematic representation of how convergent evidence implicates *RELN* silencing in schizophrenia. Epigenetic studies demonstrate *RELN* promoter hypermethylation with upregulation of *DNMT1*/*3A*, leading to reduced *RELN* transcription and protein expression. Transcriptomic and proteomic findings confirm downregulation of *RELN* and associated GABAergic signaling deficits (e.g., reduced *GAD1* expression and impaired inhibitory tone). At the cellular and circuit levels, iPSC and post-mortem studies converge on reduced inhibition and cortical network imbalance, potentially contributing to hyperexcitability. Potential therapeutic implications are suggested by experimental evidence that histone deacetylase (HDAC) inhibition (e.g., valproic acid) can counteract *RELN* promoter hypermethylation by reducing histone deacetylation, thereby promoting a more open chromatin state that facilitates transcriptional reactivation of *RELN*. This highlights one example of how mechanistic insights may guide future investigation of targeted interventions. Symbols and arrows used in this figure: solid arrows (→) indicate directional influence or process flow (not necessarily causal); up arrows (↑) indicate increase/up-regulation; down arrows (↓) indicate decrease/down-regulation; the dashed green arrow denotes a conceptual therapeutic/rescue step that alleviates repression; and the pink “lollipop” marks on DNA depict CpG DNA methylation at promoters.

Several examples illustrate how agreement across domains strengthens biological confidence. For example, *DLG4* (PSD-95), which is not a genome-wide association locus, yet it shows consistent signals. These include reduced enhancer acetylation in DLPFC, decreased transcript abundance, lower protein expression in cortical regions, and reduced puncta and EPSCs in iPSC-derived neurons, deficits that can be pharmacologically reversed. *C4A/C4B* structural variation in the MHC is another robust example. Combining genetic association, epigenomic enrichment at enhancers, transcriptomic upregulation in immune modules, and elevated serum protein levels predictive of treatment response. While convergence across molecular layers is evident in several pathways, *RELN* provides a particularly illustrative example. Epigenetic studies have consistently reported *RELN* promoter hypermethylation with *DNMT* upregulation. Transcriptomic and proteomic analyses show reduced *RELN* expression. Post-mortem cortical studies link this to impaired GABAergic signaling and reduced inhibitory tone. iPSC-derived neuronal models similarly demonstrate synaptic and network-level deficits consistent with *RELN* downregulation. Together, these findings converge on a mechanistic framework which inkins *RELN* silencing to cortical network imbalance (Figure 2). Importantly, this example illustrates how integrating evidence across genetics, epigenetics, transcriptomics, proteomics, and cellular models can move the field toward actionable mechanistic hypotheses.

Yet convergence is far from universal. Every molecular domain carries limitations. Genetic studies, while powered by large-scale GWASs, remain disproportionately biased toward European-ancestry cohorts which restricts generalizability. Epigenetic and transcriptomic studies face tissue-specific variability and difficulties in cross-cohort harmonization. iPSC models are constrained by developmental immaturity, line-to-line variability, and a lack of environmental inputs. Proteomics, in particular, has lagged behind other omics, owing to the instability of proteins in post-mortem tissue, the limited sensitivity of earlier mass spectrometry platforms, and the influence of confounders such as medication status. These challenges contribute to modest effect sizes and variable replication for candidate biomarkers like IL-6, CRP, and C4A. Encouragingly, advances in high-resolution, single-cell, and spatial proteomics are beginning to address these limitations, raising the prospect that proteomics may ultimately reach a level of resolution comparable to that of transcriptomics. Environmental influences further complicate molecular findings. Prenatal exposures such as infection, malnutrition, and hypoxia leave lasting epigenetic marks in the placenta and developing brain. Postnatal exposures, including cannabis use, urbanicity, and psychosocial trauma, interact with genetic liability to alter chromatin accessibility and transcriptional regulation. Convergence frameworks must therefore integrate not only molecular alterations but also environmental risk factors across developmental time points.

Heterogeneity is another key challenge. Subgrouping approaches reveal partially distinct biological profiles, such as synaptic-loaded versus immune-loaded signatures, and these may not generalize across all patients. Environmental exposures, developmental stage, and treatment history add further stratification. Thus, convergence may occur within subsets, but divergence may occur across the broader population. Recognizing subgroup-specific trajectories will be critical for refining convergence frameworks and guiding precision approaches that reflect the disorder’s complexity.

Computational tools are increasingly used to integrate molecular datasets, but they face substantial limitations. Many predictive models are trained on relatively small sample sizes, raising the risk of overfitting and inflated accuracy estimates. Polygenic risk scores, though informative, perform poorly in non-European populations due to differences in allele frequencies and linkage disequilibrium, limiting cross-ancestry portability. Moreover, harmonizing datasets across tissues, platforms, and pipelines remains an unresolved challenge. Addressing these issues will require larger, ancestrally diverse cohorts, standardized analytic frameworks, and rigorous validation strategies.

From a translational standpoint, convergent molecular findings are beginning to inform precision psychiatry. Integration of polygenic risk scores with transcriptomic or proteomic biomarkers may enhance prediction of treatment responsiveness, particularly for immune- or synaptic-related subgroups. iPSC-derived neurons and organoids provide functional assays of patient-specific neuronal physiology and drug response, with proof-of-principle studies already demonstrating the stratification of lithium or clozapine responders. However, no biomarker has yet achieved the robustness required for clinical implementation. Systematic validation in large, diverse cohorts and the establishment of unified clinical pipelines will be needed to move from preliminary promise toward clinical application.

Emerging evidence suggests that distinct axes of molecular convergence may map onto specific symptom clusters. For example, synaptic signaling alterations have been associated with cognitive impairment [13], whereas immune dysregulation and mitochondrial dysfunction have been linked to negative symptoms in some cohorts [58,63,64,65]. While these associations remain provisional and inconsistently replicated, they underscore the importance of investigating how biological convergence may contribute to clinical heterogeneity. Linking molecular pathways to symptom domains may enhance stratification strategies and ultimately inform targeted interventions.

## 9. Conclusions and Clinical Outlook

Our synthesis highlights recurring molecular axes, synaptic signaling, immune regulation (including complement), mitochondrial bioenergetics, neurodevelopment, and cell adhesion that together suggest the possibility of cross-level convergence in schizophrenia. Translationally, near-term opportunities include combining polygenic risk scores with transcriptomic/proteomic readouts for stratification and prognosis; however, current PRS performance and portability remain limited, especially across ancestries, and thus require cautious, quantitatively benchmarked deployment. Advances in single-cell and spatial profiling now permit cell-type-resolved maps that can anchor biomarkers and drug targets in specific neuronal and glial populations, accelerating mechanism-driven pipelines [45]. Immune-synaptic mechanisms, exemplified by C4A/C4B biology, illustrate how genetic risk can inform pathway-focused trials (e.g., complement or microglial modulators) once safety and target engagement are established [60]. Patient-derived iPSC systems, while still constrained by variability and developmental immaturity, are increasingly useful for functional genomics and pharmacology and may evolve into screening tools to anticipate drug responsiveness in defined subgroups [112]. These observations merit quantitative testing to determine whether apparent convergence reflects genuine biology or methodological focus. Preregistered, cross-cohort meta-analyses on harmonized, ancestrally diverse datasets should report calibrated effect sizes and replication across layers. With these foundations, stratified, mechanism-anchored trials become tractable. 

## Figures and Tables

**Table 1:** Classes of genetic variants implicated in schizophrenia.

Variant Class	Tissue/ Sample	Example Loci/ Genes	Frequency	Effect Size	Main Pathways	References
SNPs	Peripheral blood (germline DNA; 36,989 cases/113,075 controls)	*CACNA1C; MIR137*; *GRIN2A*	High minor allele frequency (MAF) 20–50%	Small per-allele effect: 1.06–1.12×	Synaptic signaling; calcium channel regulation	[11,16]
CNVs	Peripheral blood (germline DNA; >21,000 cases/>20,000 controls)	22q11.2 deletion; 16p11.2 duplication	Rare ~0.3%	Moderate–large 9–30×	Neurodevelopment immune modulation	[20]
Rare LoF coding mutations	Peripheral blood WES (germline DNA; 6000 cases/6000 controls)	*SETD1A*; *RBM12*; *GRIN2A*; *TRIO*; *CACNA1G*	Very rare <0.1% cases	Large (high penetrance) OR: 3–50×	Chromatin remodeling transcriptional regulation synaptogenesis; glutamatergic signaling; ion channel regulation	[13,37,77]
Regulatory eQTLs	DLPFC post-mortem (RNA-seq/eQTL; 467 donors)	Non-coding SNPs modulating *DRD2*	Common minor allele frequency (MAF) ~10–30%	Small ~1.1	Gene expression modulation	[12,13]
PRS	Peripheral blood genotyping (arrays; PRS derived from >320,000	Aggregate of 10^4^–10^6^ SNPs	Present in all ancestries	Cumulative across many loci	Pleiotropic effects on neurodevelopment and immunity	[74]

**Table 2:** Principal epigenetic and chromatin-based pathways implicated in schizophrenia.

Epigenetic Mechanism	Sample/Tissue	Key Examples	Impact on Gene Regulation	Main Pathways	References
**DNA Methylation**	– Post-mortem PFCs (BA9, n = 14 cases vs. 14 controls) – Post-mortem DLPFCs (n = 15 vs. 15) – Placentae (n = 157) – PBMCs (n = 20 vs. 20)	– *RELN* and *GAD1* promoter hypermethylation in BA9 and DLPFC – DNMT1/3A upregulation in PFC – Placental DMRs at immune (*CXCL10*, HLA) and oxidative stress loci mediating PRS × obstetric complications – *DRD2* methylation in blood	Transcriptional repression	GABAergic signaling; immune regulation	[38,88,89,90]
**Histone Modifications**	– PFC neuronal nuclei (BA9; 10 cases vs. 10 controls) – DLPFC ChIP-seq (n = 236 donors)	– ↑ H3R17me at metabolic gene promoters with concomitant mRNA downregulation – 3000+ DLPFC enhancers with altered H3K27ac – Enrichment of risk variants at combined H3K4me3 + H3K27ac peaks in excitatory neurons	Altered chromatin accessibility	Synaptic plasticity; immune response	[12,39,40]
**Chromatin Looping (3D Contacts)**	– Promoter-capture Hi-C in adult DLPFCs (3 cases, 3 controls) – In situ Hi-C in adult PFC (5 donors) – Hi-C in iPSC-derived neurons	– Schizophrenia loci contacting *GRIN2B*, *MEF2C*, and *C4A* genes – Enrichment of risk SNPs at loop anchors genome-wide – One-third of non-coding SNPs link to synaptic and chromatin-remodeling gene promoters	Long-range regulation of risk genes	Synaptic pruning; neurodevelopment	[19,40,87]
**Non-Coding RNAs (miRNA and lncRNA)**	– DLPFC tissue and paired plasma (30 cases vs. 30 controls; 60 × 60)	– ↓ miR-137 in both DLPFC and plasma, derepressing *SYN2* and *IL6R* – ↑ *NEAT1/MALAT1* lncRNAs correlated with CRP and oxidative markers – Organoid studies: miR-137 correction rescues arborization deficits	Post-transcriptional modulation; network rewiring	Neuronal differentiation; immune signaling	[87,91]
**Peripheral Epigenetic Signatures**	– PBMCs (20 antipsychotic-naïve cases vs. 20 controls) – Saliva (25 vs. 25) – Olfactory epithelium (small pilot)	– Global hypomethylation in PBMCs – *DRD2* promoter hypermethylation in blood – *ST6GALNAC1* promoter hypermethylation in saliva correlated with IL-6 (r = 0.56)	Potential peripheral biomarkers	Neuroimmune regulation; diagnostic utility	[90,91]

Table note. Arrows indicate direction of effect relative to controls: ↑ increase/up-regulation; ↓ decrease/down-regulation. The symbol × denotes an interaction term (e.g., PRS × obstetric complications), and “+” indicates co-occurrence of histone marks (e.g., H3K4me3 + H3K27ac). Gene symbols are italicized (e.g., *RELN*, *GAD1*, *GRIN2B*, *MEF2C*, *C4A*, *DRD2*, *ST6GALNAC1*, *SYN2*, *IL6R*); mature microRNAs are not italicized (e.g., miR-137). Abbreviations: BA, Brodmann area; PFC, prefrontal cortex; DLPFC, dorsolateral PFC; PBMC, peripheral blood mononuclear cell; iPSC, induced pluripotent stem cell; DMR, differentially methylated region; PRS, polygenic risk score; ChIP-seq, chromatin immunoprecipitation sequencing; Hi-C, chromosome-conformation capture; SNP, single-nucleotide polymorphism; lncRNA, long non-coding RNA; miRNA, microRNA; CRP, C-reactive protein; GABAergic, γ-aminobutyric-acid–mediated inhibitory signaling; H3K27ac, histone H3 lysine-27 acetylation; H3K4me3, histone H3 lysine-4 trimethylation; H3R17me, histone H3 arginine-17 methylation.

**Table 3:** Major transcriptomic alterations in schizophrenia.

Transcriptomic Feature	Sample/Tissue	Key Examples	Functional Impact	Main Pathways	References
**Differential Gene Expression**	DLPFC (245 schizophrenia patients vs. 279 controls); hippocampus (48 vs. 48)	245 DEGs in DLPFC; 48 DEGs in hippocampus	Altered mRNA abundance	Synaptic signaling; mitochondrial function; immune response	[13,44]
**Alternative Splicing and Isoform Shifts**	Frontal and temporal cortices (258 schizophrenia patients vs. 301 controls); DLPFC BA46 (100 vs. 100); BA10 (40 vs. 40)	3803 dysregulated isoforms; 515 splicing events (e.g., *DCLK1* and *PLP1*); disrupted exon usage in *ENAH* and *CPNE3*; many events map to eQTLs	Isoform-specific expression changes	Neurodevelopment; neurotransmission; myelination	[12,18,99,100]
**Non-Coding RNA Dysregulation**	Amygdalae (13 schizophrenia patients vs. 14 controls); LCLs (20 vs. 20); DLPFCs (258 vs. 301); PBMCs (36 vs. 15)	↓ miRNAs (miR-1307, miR-34 family, and miR-137); ↓ *DICER1* expression; lncRNA co-expression modules	Post-transcriptional regulation; network rewiring	Neuronal maturation; immune modulation	[18,98,101,102,104]
**Tissue-Specific and Peripheral Signatures**	PBMCs (36 schizophrenia patients vs. 15 controls); LCLs (20 vs. 20)	Upregulation of immune-related genes in PBMCs and LCLs	Systemic transcriptional alterations	Neuroimmune signaling; biomarker potential	[61,104]
**Co-Expression Network Perturbations**	DLPFCs (258 schizophrenia patients vs. 279 controls); frontal and temporal cortices (258 vs. 301)	Synaptic, glial, and immune modules identified by WGCNA; modules enriched for GWAS risk variants	Coordinated dysregulation of gene clusters	Synaptic transmission; glial function; immunity	[13,18,77]

Table note. Arrows indicate direction of effect relative to controls: ↓ decrease/down-regulation. Gene symbols are italicized (e.g., *DCLK1*, *PLP1*, *ENAH*, *CPNE3*, *DICER1*), while mature microRNAs are not italicized (e.g., miR-1307, miR-34 family, miR-137). Abbreviations: DLPFC, dorsolateral prefrontal cortex; BA, Brodmann area; PBMC, peripheral blood mononuclear cell; LCL, lymphoblastoid cell line; DEG, differentially expressed gene; eQTL, expression quantitative trait locus; miRNA, microRNA; lncRNA, long non-coding RNA; WGCNA, weighted gene co-expression network analysis; GWAS, genome-wide association study.

**Table 4:** Major proteomic alterations in schizophrenia.

Section	Sample/Tissue	Key Examples	Functional Impact	Main Pathways	Ref.
Synaptic proteome	Anterior cingulate cortex (ACC; 20 SZ vs. 20 CTRLs)	Differential PSD proteins (e.g., DNM1, AP2B1)	Vesicle-cycling/plasticity changes	Synaptic signaling	[7]
	Primary auditory cortex (A1; 48 SZ vs. 48 CTRLs)	↓ PSD markers (PSD-95/SHANK3) confined to synaptic fraction	Synaptic dysfunction; altered vesicle/plasticity machinery	Synaptic signaling	[48]
Mitochondrial/energetic proteome	DLPFC/ACC cortex (post-mortem)	(post-mortem) Altered respiratory-chain (Complex I–V) and energy-metabolism proteins	Synaptic–energetic coupling deficits; cellular energetics changes	Mitochondrial OXPHOS; metabolic pathways	[46]
Immune and Inflammatory Markers	Serum (multi-cohort case–control)	34-analyte serum signature distinguishing SZ from controls/other disorders; cross-cohort classification (∼60–75%)	Systemic immune activation; diagnostic signal	Cytokine, growth-factor and endocrine networks	[49]
	Serum (first-episode, antipsychotic-naïve; sex-stratified)	Sex-specific molecular profiles (16 molecules differing by sex across four cohorts)	Hormonal/inflammatory dysregulation; sex effects	Immune & endocrine biomarkers	[108]
	Plasma (large discovery/replication)	Multi-analyte plasma panel distinguishing SZ and depression in large collections	Diagnostic stratification (discovery + validation)	Immune/inflammatory & growth-factor networks	[50]
	Trial cohort (OPTiMiSE)	Complement-pathway changes in blood proteomics; explored as predictors of antipsychotic response	Treatment-response prediction signal	Complement activation	[62]
Post-Translational Modifications	Serum phosphoproteome (50 SZ vs. 50 CTRL)	Altered phosphorylation of signaling and acute-phase proteins (e.g., Akt1/STAT3 pathways; coagulation/synaptic scaffolding sites)	Dysregulated signaling; immune–coagulation crosstalk	Protein phosphorylation; signal transduction	[52]

Table note. Symbols: ↓ decrease/down-regulation. Abbreviations: SZ, schizophrenia; CTRL(s), control(s); ACC, anterior cingulate cortex; A1, primary auditory cortex; DLPFC, dorsolateral prefrontal cortex; PSD, postsynaptic density; OXPHOS, oxidative phosphorylation (electron-transport chain complexes I–V that generate ATP). Per IJMS/MDPI policy, define abbreviations at first use in the abstract, main text, and in the first figure/table where they appear.

**Table 5:** Key findings from iPSC models of schizophrenia.

Main Pathways	Model/System and Format	Cohort (Schizophrenia Patients vs. CTRLs)	Key Molecular Findings	Functional Consequence	References
**Neurodevelopment and Transcriptional Dysregulation**	Forebrain NPCs (2D culture)	4 schizophrenia patients vs. 4 CTRLs	↓ NCAM1/NRXN1/NLGN1 (1.5–1.7×), ↑ antioxidant enzymes (2.2×); miR-137 ↑ 1.8×, miR-9 ↓ 1.4×; SOX2 ↓ 40%, PAX6 ↓ 30%	Migration −35%, ROS +28%, MAP2 onset delayed ~7 d	[53,66]
**Mitochondrial Dysfunction and Oxidative Stress**	2D neurons (dopaminergic and glutamatergic) and 3D organoids	3 schizophrenia patients vs. 2 CTRLs (neurons); 8 schizophrenia patients vs. 8 CTRLs (organoids)	Mito fragmentation +30%; ΔΨm −25%; ROS +35%Basal OCR −22%; ATP-linked OCR −28%	Neurite length −20%; spike rate −40%	[58]
**Synaptic Connectivity and Dendritic Architecture**	2D cortical neurons	4 schizophrenia patients (incl. 22q11.2del) vs. 3 CTRLs	PSD-95 puncta −40%; dendritic intersections −35%; OCT4/NANOG persistence; Syn1 ↓ 32%, NRXN1↓28%	sEPSC frequency −50%; loxapine rescue PSD-95 +25%; EPSC +30%	[29,59]
**Circuit-Level Vulnerabilities**	Interneuron co-cultures (2D) and cerebral organoids (3D)	9 schizophrenia patients vs. 9 CTRLs (interneurons); 9 schizophrenia patients vs. 5 CTRLs (organoids; n = 25)	VGAT^+^ puncta −30%; GAD67 −42%; gephyrin −38%; NLGN2 −45%BRN2 −50%; PTN −60%	Firing rate rescue +50% (NLGN2/NAC); progenitor survival +40%, NeuN^+^ neurons ×2 (PTN)	[55,57]
**Oligodendrocyte Precursor Dysfunction**	NG2^+^ OPCs (2D culture)	3 CSPG4-mut schizophrenia patients vs. 3 siblings	NG2 high-mannose ×3; MBP −45%; PLP1 −50%; SOX10/OLIG2 −30%	In vivo FA −15% (DTI)	[54]

Table note. Symbols indicate direction/magnitude relative to controls: ↑ increase/upregulation; ↓ decrease/downregulation; + increase (absolute); − decrease (absolute); × fold (“times”); Δ change from baseline. Gene symbols. Gene symbols are italicized (e.g., *NCAM1*, *NRXN1*, *NLGN1*, *SOX2*, *PAX6*, *MAP2*, *NLGN2*, *POU3F2*/*BRN2*, *PTN*, *MBP*, *PLP1*, *SOX10*, *OLIG2*). Abbreviations. DLPFC, dorsolateral prefrontal cortex; DTI, diffusion tensor imaging; EPSC/sEPSC, (spontaneous) excitatory postsynaptic current; FA, fractional anisotropy; GAD67, 67-kDa glutamate decarboxylase (product of *GAD1*); iPSC, induced pluripotent stem cell; MBP, myelin basic protein; NAC, N-acetylcysteine; NeuN, neuronal nuclei antigen (*RBFOX3*); NG2, chondroitin sulfate proteoglycan 4 (*CSPG4*); NPC, neural progenitor cell; OCR, oxygen consumption rate; OPC, oligodendrocyte precursor cell; PFC, prefrontal cortex; PSD-95, postsynaptic density protein 95; ΔΨm, mitochondrial membrane potential; VGAT, vesicular GABA transporter (*SLC32A1*).

**Table 6:** Convergent molecular pathways across five data layers.

Pathway	Genetics	Epigenetics	Transcriptomics	Proteomics	iPSC Models
**Synaptic Signaling**	*CACNA1C*, *GRIN2A*, and *DLG2* GWAS loci [11,16]; *SETD1A* LoF [37]	↓ H3K27ac/H3K4me3 at synaptic enhancers [12,40]	Disrupted synaptic co-expression modules; isoform shifts [13,18]	↓ PSD-95/SHANK3; phospho-Akt1 alterations [47,48,52]	↓ PSD-95 puncta and sEPSCs; loxapine rescue [29,55]
**Mitochondrial Bioenergetics**	Mito-ETC gene variants; 16p11.2 CNV [20,21]	H3R17me at metabolic promoters [39]	Downregulated *OXPHOS* transcripts [44]	↓ Complex I–V subunits; altered mitochondrial proteins [47]	Mito fragmentation, ↓ ΔΨm, ↑ ROS [58]; ↓ OCR in organoids [56]
**Cell-Adhesion Complexes**	*NRXN1*/*NLGN1* CNVs; *NCAM1*/*BRN2* risk loci [11,20]	*RELN* promoter hypermethylation [88,89]	Dysregulated protocadherins and adhesion isoforms [18]	Altered AP2B1/DNM1 phosphorylation [48,109]	↓ *NCAM1*/*NRXN1*/*NLGN1* in NPCs [53]; OPC NG2 misprocessing [54]
**Immune Regulation**	*C4A*/*C4B* MHC variation [60]	Placental/blood immune-gene DMRs [38,90]	Upregulated cytokine/microglial modules [18,104]	IL-6, CFI, and C4A serum signatures [49,62]	Rescue of complement/cytokine defects by PTN or NAC [57]
**Neurodevelopmental Regulation**	22q11.2del; *POU3F2/BRN2, PTN* risk loci [57]	Placental DMRs at *PAX6/SOX2*; developmental histone marks [38]	Disrupted NPC and neuronal differentiation modules [53,66]	↓ BRN2, PTN in organoid proteomes [57]	NPC migration delays; miR-137/PAX6 imbalance; organoid progenitor loss [53,66]

Table note. Symbols indicate direction relative to controls: ↓ decrease/down-regulation; ↑ increase/up-regulation. Gene symbols are italicized (e.g., *CACNA1C*, *GRIN2A*, *DLG2*, *SETD1A*, *NRXN1*, *NLGN1*, *NCAM1*, *RELN*, *C4A/C4B*, *POU3F2*, *PTN*, *PAX6*, *SOX2*); proteins remain roman (e.g., PSD-95, SHANK3, Akt1). “phospho-” denotes phosphorylated protein (e.g., phospho-Akt1). Complex I–V refer to mitochondrial electron-transport chain complexes. Abbreviations: GWAS, genome-wide association study; LoF, loss-of-function; CNV, copy-number variant; DMR, differentially methylated region; OXPHOS, oxidative phosphorylation; ΔΨm, mitochondrial membrane potential; ROS, reactive oxygen species; OCR, oxygen-consumption rate; NPC, neural progenitor cell; iPSC, induced pluripotent stem cell; OPC, oligodendrocyte precursor cell; MHC, major histocompatibility complex; sEPSC, spontaneous excitatory postsynaptic current.

**Table 7:** Cross-layer “convergence” genes/proteins repeatedly implicated in schizophrenia.

Gene/Protein	Genetics	Epigenetics/Chromatin	Transcriptomics	Proteomics	iPSC Models
***DLG4* (PSD-95)**	(Not a GWAS hit)	↓ H3K27ac at *DLG4* enhancer in DLPFC [12]	↓ *DLG4* mRNA in DLPFC [13]	↓ PSD-95 in ACC and A1 cortices [47,48]	↓ PSD-95 puncta and sEPSC frequency in cortical neurons; rescued by loxapine [29]
**C4A/C4B**	Complex structural variation in MHC confers risk [60]	Enriched H3K4me3/H3K27ac at C4 loci in neurons [40]	↑ *C4A* within immune co-expression modules [18]	↑ Serum C4A/C4B in treatment responders [62]	—
***NRXN1/NLGN1***	*NRXN1* deletions and *NLGN1* GWAS signals [20]	—	↓ *NRXN1* and *NLGN1* transcripts in NPCs [53]	—	↓ Presynaptic puncta in cortical [29] and glutamatergic neurons [55,59]
***MT-CO1/ATP5A1***	Rare variants in ETC genes; 16p11.2 CNV [20,21]	—	↓ OXPHOS transcripts in cortex [44]	↓ Complex I–V subunits in ACC [47]	↑ Mitochondrial fragmentation, ↓ ΔΨm, ↑ ROS in neurons [58]
***RELN***	—	Promoter hypermethylation in BA9 [88,89]	↓ *RELN* mRNA in DLPFC [88,89]	—	VPA restores H3K9ac at *RELN* promoter and increases mRNA [93]
***POU3F2* (BRN2)/PTN**	*POU3F2*/PTN risk loci in schizophrenia organoids [57]	Placental DMRs at PAX6/SOX2, altered developmental histone marks [38]	Disrupted NPC and neuronal differentiation modules [53,66]	↓ BRN2 and PTN proteins in organoids [57]	Exogenous PTN or BRN2 rescues progenitor survival and neuronal output [57]

Table note. Symbols indicate direction relative to controls: ↓ decrease/down-regulation; ↑ increase/up-regulation; × “times/fold”; Δ change from baseline. Gene symbols are italicized (e.g., *DLG4*, *C4A/C4B*, *NRXN1*, *NLGN1*, *MT-CO1*, *ATP5A1*, *RELN*, *POU3F2*, *PTN*); proteins remain roman (e.g., PSD-95, Akt1). Abbreviations: GWAS, genome-wide association study; LoF, loss-of-function; MHC, major histocompatibility complex; CNV, copy-number variant; DMR, differentially methylated region; DLPFC, dorsolateral prefrontal cortex; ACC, anterior cingulate cortex; A1, primary auditory cortex; NPC, neural progenitor cell; iPSC, induced pluripotent stem cell; sEPSC, spontaneous excitatory postsynaptic current; OXPHOS, oxidative phosphorylation; ETC, electron-transport chain; ΔΨm, mitochondrial membrane potential; ROS, reactive oxygen species; H3K27ac/H3K4me3/H3K9ac, histone H3 lysine-27 acetylation/lysine-4 trimethylation/lysine-9 acetylation. Genes/proteins listed met a ≥3-layer support heuristic (genetic, epigenetic, transcriptomic, proteomic, and/or iPSC). Symbols denote direction versus controls: ↓ decrease; ↑ increase; × fold-change; Δ change from baseline. Abbreviations as in Table 6.

## Data Availability

No new data were created or analyzed in this study. Data sharing is not applicable to this article.

## References

[1] Rubeša G., Gudelj L., Kubinska N. (2011). Etiology of schizophrenia and therapeutic options. Psychiatr. Danub..

[2] Onaolapo A., Onaolapo O. (2018). Schizophrenia Aetiology and Drug Therapy: A Tale of Progressive Demystification and Strides in Management. Adv. Pharmacol. Pharm..

[3] Hilker R., Helenius D., Fagerlund B., Skytthe A., Christensen K., Werge T.M., Nordentoft M., Glenthøj B. (2018). Heritability of Schizophrenia and Schizophrenia Spectrum Based on the Nationwide Danish Twin Register. Biol. Psychiatry.

[4] Hyllested A. (1996). Schizophrenia. Current biological theories on the etiology. Ugeskr. Laeger.

[5] Walker E., Mittal V., Tessner K. (2008). Stress and the hypothalamic pituitary adrenal axis in the developmental course of schizophrenia. Annu. Rev. Clin. Psychol..

[6] Pandarakalam J.P. (2015). The Autoimmune and Infectious Etiological Factors of a Subset of Schizophrenia. Br. J. Med. Pract..

[7] English J.A., Fan Y., Föcking M., Lopez L.M., Hryniewiecka M., Wynne K., Dicker P., Matigian N., Cagney G., Mackay-Sim A. (2015). Reduced protein synthesis in schizophrenia patient-derived olfactory cells. Transl. Psychiatry.

[8] Obiols J.E., Vicens-Vilanova J. (2003). Etiología y Signos de Riesgo en la Esquizofrenia. Int. J. Psychol. Psychol. Ther..

[9] Insel T.R. (2010). Rethinking schizophrenia. Nature.

[10] Kahn R.S., Sommer I.E., Murray R.M., Meyer-Lindenberg A., Weinberger D.R., Cannon T.D., O’Donovan M., Correll C.U., Kane J.M., van Os J. (2015). Schizophrenia. Nat. Rev. Dis. Primers.

[11] Ripke S., Neale B.M., Corvin A., Walters J.T.R., Farh K.-H., Holmans P.A., Lee P., Bulik-Sullivan B., Collier D.A., Huang H. (2014). Biological insights from 108 schizophrenia-associated genetic loci. Nature.

[12] Jaffe A.E., Gao Y., Deep-Soboslay A., Tao R., Hyde T.M., Weinberger D.R., Kleinman J.E. (2016). Mapping DNA methylation across development, genotype and schizophrenia in the human frontal cortex. Nat. Neurosci..

[13] Fromer M., Roussos P., Sieberts S.K., Johnson J.S., Kavanagh D.H., Perumal T.M., Ruderfer D.M., Oh E.C., Topol A., Shah H.R. (2016). Gene expression elucidates functional impact of polygenic risk for schizophrenia. Nat. Neurosci..

[14] Cariaga-Martinez A., Saiz-Ruiz J., Alelú-Paz R. (2016). From Linkage Studies to Epigenetics: What We Know and What We Need to Know in the Neurobiology of Schizophrenia. Front. Neurosci..

[15] Dempster E., Viana J., Pidsley R., Mill J. (2012). Epigenetic Studies of Schizophrenia: Progress, Predicaments, and Promises for the Future. Schizophr. Bull..

[16] Trubetskoy V., Pardiñas A., Qi T., Panagiotaropoulou G., Awasthi S., Bigdeli T., Bryois J., Chen C.-Y., Dennison C., Hall L. (2022). Mapping genomic loci implicates genes and synaptic biology in schizophrenia. Nature.

[17] Hoffman G.E., Hartley B.J., Flaherty E., Ladran I., Gochman P., Ruderfer D.M., Stahl E.A., Rapoport J., Sklar P., Brennand K.J. (2017). Transcriptional signatures of schizophrenia in hiPSC-derived NPCs and neurons are concordant with post-mortem adult brains. Nat. Commun..

[18] Gandal M.J., Zhang P., Hadjimichael E., Walker R.L., Chen C., Liu S., Won H., van Bakel H., Varghese M., Wang Y. (2018). Transcriptome-wide isoform-level dysregulation in ASD, schizophrenia, and bipolar disorder. Science.

[19] Wang D., Liu S., Warrell J., Won H., Shi X., Navarro F.C.P., Clarke D., Gu M., Emani P., Yang Y.T. (2018). Comprehensive functional genomic resource and integrative model for the human brain. Science.

[20] Malhotra D., Sebat J. (2012). CNVs: Harbingers of a Rare Variant Revolution in Psychiatric Genetics. Cell.

[21] Sullivan P.F., Daly M.J., O’Donovan M. (2012). Genetic architectures of psychiatric disorders: The emerging picture and its implications. Nat. Rev. Genet..

[22] Borgmann-Winter K.E., Wang K., Bandyopadhyay S., Torshizi A.D., Blair I.A., Hahn C.G. (2020). The proteome and its dynamics: A missing piece for integrative multi-omics in schizophrenia. Schizophr. Res..

[23] Nascimento J.M., Martins-de-Souza D. (2015). The proteome of schizophrenia. npj Schizophr..

[24] Guan F., Ni T., Zhu W., Williams L.K., Cui L.-B., Li M., Tubbs J., Sham P.-C., Gui H. (2022). Integrative omics of schizophrenia: From genetic determinants to clinical classification and risk prediction. Mol. Psychiatry.

[25] Liu J., Chen J., Perrone-Bizzozero N., Calhoun V.D. (2018). A Perspective of the Cross-Tissue Interplay of Genetics, Epigenetics, and Transcriptomics, and Their Relation to Brain Based Phenotypes in Schizophrenia. Front. Genet..

[26] Burrack N., Yitzhaky A., Mizrahi L., Wang M., Stern S., Hertzberg L. (2024). Altered Expression of PDE4 Genes in Schizophrenia: Insights from a Brain and Blood Sample Meta-Analysis and iPSC-Derived Neurons. Genes.

[27] Stern S., Zhang L., Wang M., Wright R., Rosh I., Hussein Y., Stern T., Choudhary A., Tripathi U., Reed P. (2024). Monozygotic twins discordant for schizophrenia differ in maturation and synaptic transmission. Mol. Psychiatry.

[28] Sarkar A., Mei A., Paquola A.C.M., Stern S., Bardy C., Klug J.R., Kim S., Neshat N., Kim H.J., Ku M. (2018). Efficient Generation of CA3 Neurons from Human Pluripotent Stem Cells Enables Modeling of Hippocampal Connectivity In Vitro. Cell Stem Cell.

[29] Brennand K.J., Simone A., Jou J., Gelboin-Burkhart C., Tran N., Sangar S., Li Y., Mu Y., Chen G., Yu D. (2011). Modelling schizophrenia using human induced pluripotent stem cells. Nature.

[30] Romanovsky E., Choudhary A., Peles D., Abu-Akel A., Stern S. (2025). Uncovering convergence and divergence between autism and schizophrenia using genomic tools and patients’ neurons. Mol. Psychiatry.

[31] Birnbaum R., Weinberger D.R. (2017). Genetic insights into the neurodevelopmental origins of schizophrenia. Nat. Rev. Neurosci..

[32] Mizrahi L., Choudhary A., Ofer P., Goldberg G., Milanesi E., Kelsoe J.R., Gurwitz D., Alda M., Gage F.H., Stern S. (2023). Immunoglobulin genes expressed in lymphoblastoid cell lines discern and predict lithium response in bipolar disorder patients. Mol. Psychiatry.

[33] Sharma O., Nayak R., Mizrahi L., Rike W.A., Choudhary A., Hussein Y., Rosh I., Tripathi U., Shemen A., Squassina A. (2024). Predicting Suicide Risk in Bipolar Disorder patients from Lymphoblastoid Cell Lines genetic signatures. bioRxiv.

[34] Stern S., Linker S., Vadodaria K.C., Marchetto M.C., Gage F.H. (2018). Prediction of response to drug therapy in psychiatric disorders. Open Biol..

[35] Marshall C.R., Howrigan D.P., Merico D., Thiruvahindrapuram B., Wu W., Greer D.S., Antaki D., Shetty A., Holmans P.A., Pinto D. (2017). Contribution of copy number variants to schizophrenia from a genome-wide study of 41,321 subjects. Nat. Genet..

[36] Purcell S.M., Moran J.L., Fromer M., Ruderfer D., Solovieff N., Roussos P., O’Dushlaine C., Chambert K., Bergen S.E., Kähler A. (2014). A polygenic burden of rare disruptive mutations in schizophrenia. Nature.

[37] Singh T., Poterba T., Curtis D., Akil H., Al Eissa M., Barchas J.D., Bass N., Bigdeli T.B., Breen G., Bromet E.J. (2022). Rare coding variants in ten genes confer substantial risk for schizophrenia. Nature.

[38] Ursini G., Punzi G., Chen Q., Marenco S., Robinson J.F., Porcelli A., Hamilton E.G., Mitjans M., Maddalena G., Begemann M. (2018). Convergence of placenta biology and genetic risk for schizophrenia. Nat. Med..

[39] Akbarian S., Ruehl M.G., Bliven E., Luiz L.A., Peranelli A.C., Baker S.P., Roberts R.C., Bunney W.E., Conley R.C., Jones E.G. (2005). Chromatin Alterations Associated With Down-regulated Metabolic Gene Expression in the Prefrontal Cortex of Subjects With Schizophrenia. Arch. Gen. Psychiatry.

[40] Gusev F.E., Reshetov D.A., Mitchell A.C., Andreeva T.V., Dincer A., Grigorenko A.P., Fedonin G., Halene T., Aliseychik M., Filippova E. (2019). Chromatin profiling of cortical neurons identifies individual epigenetic signatures in schizophrenia. Transl. Psychiatry.

[41] Girdhar K., Hoffman G.E., Jiang Y., Brown L., Kundakovic M., Hauberg M.E., Francoeur N.J., Wang Y.-c., Shah H., Kavanagh D.H. (2018). Cell-specific histone modification maps in the human frontal lobe link schizophrenia risk to the neuronal epigenome. Nat. Neurosci..

[42] Won H., de la Torre-Ubieta L., Stein J.L., Parikshak N.N., Huang J., Opland C.K., Gandal M.J., Sutton G.J., Hormozdiari F., Lu D. (2016). Chromosome conformation elucidates regulatory relationships in developing human brain. Nature.

[43] Hoffman G.E., Bendl J., Girdhar K., Schadt E.E., Roussos P. (2019). Functional interpretation of genetic variants using deep learning predicts impact on chromatin accessibility and histone modification. Nucleic Acids Res..

[44] Collado-Torres L., Burke E.E., Peterson A., Shin J., Straub R.E., Rajpurohit A., Semick S.A., Ulrich W.S., Price A.J., Valencia C. (2019). Regional Heterogeneity in Gene Expression, Regulation, and Coherence in the Frontal Cortex and Hippocampus across Development and Schizophrenia. Neuron.

[45] Ruzicka W.B., Mohammadi S., Fullard J.F., Davila-Velderrain J., Subburaju S., Tso D.R., Hourihan M., Jiang S., Lee H.-C., Bendl J. (2024). Single-cell multi-cohort dissection of the schizophrenia transcriptome. Science.

[46] Pennington K., Beasley C.L., Dicker P., Fagan A., English J., Pariante C.M., Wait R., Dunn M.J., Cotter D.R. (2008). Prominent synaptic and metabolic abnormalities revealed by proteomic analysis of the dorsolateral prefrontal cortex in schizophrenia and bipolar disorder. Mol. Psychiatry.

[47] Föcking M., Lopez L.M., English J.A., Dicker P., Wolff A., Brindley E., Wynne K., Cagney G., Cotter D.R. (2014). Proteomic and genomic evidence implicates the postsynaptic density in schizophrenia. Mol. Psychiatry.

[48] MacDonald M.L., Garver M., Newman J., Sun Z., Kannarkat J., Salisbury R., Glausier J., Ding Y., Lewis D.A., Yates N. (2020). Synaptic Proteome Alterations in the Primary Auditory Cortex of Individuals With Schizophrenia. JAMA Psychiatry.

[49] Schwarz E., Guest P.C., Rahmoune H., Harris L.W., Wang L., Leweke F.M., Rothermundt M., Bogerts B., Koethe D., Kranaster L. (2012). Identification of a biological signature for schizophrenia in serum. Mol. Psychiatry.

[50] Domenici E., Willé D.R., Tozzi F., Prokopenko I., Miller S., McKeown A., Brittain C., Rujescu D., Giegling I., Turck C.W. (2010). Plasma protein biomarkers for depression and schizophrenia by multi analyte profiling of case-control collections. PLoS ONE.

[51] Rodrigues J.E., Martinho A., Santa C., Madeira N., Coroa M., Santos V., Martins M.J., Pato C.N., Macedo A., Manadas A. (2022). Systematic Review and Meta-Analysis of Mass Spectrometry Proteomics Applied to Human Peripheral Fluids to Assess Potential Biomarkers of Schizophrenia. Int. J. Mol. Sci..

[52] Jaros J.A.J., Martins-de-Souza D., Rahmoune H., Rothermundt M., Leweke F.M., Guest P.C., Bahn S. (2012). Protein phosphorylation patterns in serum from schizophrenia patients and healthy controls. J. Proteom..

[53] Brennand K., Savas J.N., Kim Y., Tran N., Simone A., Hashimoto-Torii K., Beaumont K.G., Kim H.J., Topol A., Ladran I. (2014). Phenotypic differences in hiPSC NPCs derived from patients with schizo phrenia. Mol. Psychiatry.

[54] de Vrij F.M., Bouwkamp C.G., Gunhanlar N., Shpak G., Lendemeijer B., Baghdadi M., Gopalakrishna S., Ghazvini M., Li T.M., Quadri M. (2019). Candidate CSPG4 mutations and induced pluripotent stem cell modeling implicate oligodendrocyte progenitor cell dysfunction in familial schizophrenia. Mol. Psychiatry.

[55] Kathuria A., Lopez-Lengowski K., Watmuff B., McPhie D., Cohen B.M., Karmacharya R. (2019). Synaptic deficits in iPSC-derived cortical interneurons in schizophrenia are mediated by NLGN2 and rescued by N-acetylcysteine. Transl. Psychiatry.

[56] Kathuria A., Lopez-Lengowski K., Jagtap S.S., McPhie D., Perlis R.H., Cohen B.M., Karmacharya R. (2020). Transcriptomic Landscape and Functional Characterization of Induced Pluripotent Stem Cell–Derived Cerebral Organoids in Schizophrenia. JAMA Psychiatry.

[57] Notaras M., Lodhi A., Dündar F., Collier P., Sayles N.M., Tilgner H., Greening D., Colak D. (2022). Schizophrenia is defined by cell-specific neuropathology and multiple neurodevelopmental mechanisms in patient-derived cerebral organoids. Mol. Psychiatry.

[58] Robicsek O., Karry R., Petit I., Salman-Kesner N., Müller F.J., Klein E., Aberdam D., Ben-Shachar D. (2013). Abnormal neuronal differentiation and mitochondrial dysfunction in hair follicle-derived induced pluripotent stem cells of schizophrenia patients. Mol. Psychiatry.

[59] Pedrosa E., Sandler V., Shah A., Carroll R., Chang C., Rockowitz S., Guo X., Zheng D., Lachman H.M. (2011). Development of Patient-Specific Neurons in Schizophrenia Using Induced Pluripotent Stem Cells. J. Neurogenet..

[60] Sekar A., Bialas A.R., de Rivera H., Davis A., Hammond T.R., Kamitaki N., Tooley K., Presumey J., Baum M., Van Doren V. (2016). Schizophrenia risk from complex variation of complement component 4. Nature.

[61] Sanders A.R., Drigalenko E.I., Duan J., Moy W., Freda J., Göring H.H.H., Gejman P.V. (2017). Transcriptome sequencing study implicates immune-related genes differentially expressed in schizophrenia: New data and a meta-analysis. Transl. Psychiatry.

[62] Föcking M., Pollak T., Dicker P., Cagney G., Winter I., Kahn R., McGuire P., Cotter D. (2018). O1.7. PROTEOMIC ANALYSIS OF BLOOD BASED SAMPLES FROM THE OPTiMiSE (OPTIMIZATION OF TREATMENT AND MANAGEMENT OF SCHIZOPHRENIA IN EUROPE) STUDY POINT TOWARDS COMPLEMENT PATHWAY PROTEIN CHANGES. Schizophr. Bull..

[63] Upthegrove R., Manzanares-Teson N., Barnes N.M. (2014). Cytokine function in medication-naive first episode psychosis: A systematic review and meta-analysis. Schizophr. Res..

[64] Khandaker G.M., Pearson R.M., Zammit S., Lewis G., Jones P.B. (2014). Association of Serum Interleukin 6 and C-Reactive Protein in Childhood With Depression and Psychosis in Young Adult Life: A Population-Based Longitudinal Study. JAMA Psychiatry.

[65] Prabakaran S., Swatton J.E., Ryan M.M., Huffaker S.J., Huang J.-J., Griffin J.L., Wayland M., Freeman T., Dudbridge F., Lilley K.S. (2004). Mitochondrial dysfunction in schizophrenia: Evidence for compromised brain metabolism and oxidative stress. Mol. Psychiatry.

[66] Ahmad R., Sportelli V., Ziller M., Spengler D., Hoffmann A. (2018). Tracing Early Neurodevelopment in Schizophrenia with Induced Pluripote nt Stem Cells. Cells.

[67] Kato H., Kimura H., Kushima I., Takahashi N., Aleksic B., Ozaki N. (2023). The genetic architecture of schizophrenia: Review of large-scale genetic studies. J. Hum. Genet..

[68] Yang H., Sun W., Li J., Zhang X. (2025). Epigenetics factors in schizophrenia: Future directions for etiologic and therapeutic study approaches. Ann. Gen. Psychiatry.

[69] Kumar R., Kumar V., Kumar A., Rana S.S., Priyanka (2025). Deciphering transcriptomic signatures in schizophrenia, bipolar disorder, and major depressive disorder. Front. Psychiatry.

[70] Hong Y., Yang Q., Song H., Ming G.L. (2023). Opportunities and limitations for studying neuropsychiatric disorders using patient-derived induced pluripotent stem cells. Mol. Psychiatry.

[71] Owen M.J., Sawa A., Mortensen P.B. (2016). Schizophrenia. Lancet.

[72] Shifman S., Johannesson M., Bronstein M., Chen S.X., Collier D.A., Craddock N.J., Kendler K.S., Li T., O’Donovan M., O’Neill F.A. (2008). Genome-Wide Association Identifies a Common Variant in the Reelin Gene That Increases the Risk of Schizophrenia Only in Women. PLoS Genet..

[73] Purcell S.M., Wray N.R., Stone J.L., Visscher P.M., O’Donovan M.C., Sullivan P.F., Sklar P., Purcell S.M., Stone J.L., Sullivan P.F. (2009). Common polygenic variation contributes to risk of schizophrenia and bipolar disorder. Nature.

[74] Legge S.E., Santoro M.L., Periyasamy S., Okewole A., Arsalan A., Kowalec K. (2021). Genetic architecture of schizophrenia: A review of major advancements. Psychol. Med..

[75] Lam M., Chen C.Y., Li Z., Martin A.R., Bryois J., Ma X., Gaspar H., Ikeda M., Benyamin B., Brown B.C. (2019). Comparative genetic architectures of schizophrenia in East Asian and European populations. Nat. Genet..

[76] Ruan Y., Lin Y.-F., Feng Y.-C.A., Chen C.-Y., Lam M., Guo Z., He L., Sawa A., Martin A.R., Stanley Global Asia Initiatives (2022). Improving polygenic prediction in ancestrally diverse populations. Nat. Genet..

[77] Pardiñas A.F., Holmans P., Pocklington A.J., Escott-Price V., Ripke S., Carrera N., Legge S.E., Bishop S., Cameron D., Hamshere M.L. (2018). Common schizophrenia alleles are enriched in mutation-intolerant genes and in regions under strong background selection. Nat. Genet..

[78] Townsley K.G., Li A., Deans P.J.M., Fullard J.F., Yu A., Cartwright S., Zhang W., Wang M., Voloudakis G., Girdhar K. (2022). Convergent impact of schizophrenia risk genes. bioRxiv.

[79] Roussos P., Katsel P., Davis K.L., Siever L.J., Haroutunian V. (2012). A System-Level Transcriptomic Analysis of Schizophrenia Using Postmortem Brain Tissue Samples. Arch. Gen. Psychiatry.

[80] Arnedo J., Svrakic D.M., Val C.d., Romero-Zaliz R., Hernández-Cuervo H., Fanous A.H., Pato M.T., Pato C.N., Erausquin G.A.d., Molecular Genetics of Schizophrenia Consortium (2015). Uncovering the Hidden Risk Architecture of the Schizophrenias: Confirmation in Three Independent Genome-Wide Association Studies. Am. J. Psychiatry.

[81] Migdalska-Richards A., Mill J. (2019). Epigenetic studies of schizophrenia: Current status and future directions. Curr. Opin. Behav. Sci..

[82] Roth T.L., Lubin F.D., Sodhi M., Kleinman J.E. (2009). Epigenetic mechanisms in schizophrenia. Biochim. Biophys. Acta (BBA)-Gen. Subj..

[83] Svrakic D.M., Zorumski C.F., Svrakic N.M., Zwir I., Cloninger C.R. (2013). Risk architecture of schizophrenia: The role of epigenetics. Curr. Opin. Psychiatry.

[84] Meyer U., Feldon J. (2010). Epidemiology-driven neurodevelopmental animal models of schizophrenia. Prog. Neurobiol..

[85] Brown A.S., Derkits E.J. (2010). Prenatal Infection and Schizophrenia: A Review of Epidemiologic and Translational Studies. Am. J. Psychiatry.

[86] Bale T.L., Baram T.Z., Brown A.S., Goldstein J.M., Insel T.R., McCarthy M.M., Nemeroff C.B., Reyes T.M., Simerly R.B., Susser E.S. (2010). Early Life Programming and Neurodevelopmental Disorders. Biol. Psychiatry.

[87] Punzi G., Bharadwaj R., Ursini G. (2018). Neuroepigenetics of Schizophrenia. Prog. Mol. Biol. Transl. Sci..

[88] Costa E., Dong E., Grayson D.R., Guidotti A., Ruzicka W., Veldic M. (2007). Reviewing the role of DNA (cytosine-5) methyltransferase overexpression in the cortical GABAergic dysfunction associated with psychosis vulnerability. Epigenetics.

[89] Gavin D.P., Sharma R.P. (2010). Histone modifications, DNA methylation, and Schizophrenia. Neurosci. Biobehav. Rev..

[90] Nishioka M., Bundo M., Kasai K., Iwamoto K. (2012). DNA methylation in schizophrenia: Progress and challenges of epigenetic studies. Genome Med..

[91] Chen Q., Li D., Jin W., Shi Y., Li Z., Ma P., Sun J., Chen S., Li P., Lin P. (2021). Research Progress on the Correlation Between Epigenetics and Schizophrenia. Front. Neurosci..

[92] Gavin D.P., Kartan S., Chase K., Grayson D.R., Sharma R.P. (2008). Reduced baseline acetylated histone 3 levels, and a blunted response to HDAC inhibition in lymphocyte cultures from schizophrenia subjects. Schizophr. Res..

[93] Guidotti A., Dong E., Kundakovic M., Satta R., Grayson D.R., Costa E. (2009). Characterization of the action of antipsychotic subtypes on valproate-induced chromatin remodeling. Trends Pharmacol. Sci..

[94] Covington H.E., Maze I., LaPlant Q.C., Vialou V.F., Ohnishi Y.N., Berton O., Fass D.M., Renthal W., Rush A.J., Wu E.Y. (2009). Antidepressant Actions of Histone Deacetylase Inhibitors. J. Neurosci..

[95] Simonini M.V., Camargo L.M., Dong E., Maloku E., Veldic M., Costa E., Guidotti A. (2006). The benzamide MS-275 is a potent, long-lasting brain region-selective inhibitor of histone deacetylases. Proc. Natl. Acad. Sci. USA.

[96] Day J.J., Sweatt J.D. (2011). Epigenetic Mechanisms in Cognition. Neuron.

[97] Nestler E.J., Peña C.J., Kundakovic M., Mitchell A., Akbarian S. (2016). Epigenetic Basis of Mental Illness. Neurosci..

[98] Liu Y., Chang X., Hahn C.G., Gur R.E., Sleiman P.A.M., Hakonarson H. (2018). Non-coding RNA dysregulation in the amygdala region of schizophrenia patients contributes to the pathogenesis of the disease. Transl. Psychiatry.

[99] Wu J.Q., Wang X., Beveridge N.J., Tooney P.A., Scott R.J., Carr V.J., Cairns M.J. (2012). Transcriptome Sequencing Revealed Significant Alteration of Cortical Promoter Usage and Splicing in Schizophrenia. PLoS ONE.

[100] Cohen O.S., McCoy S.Y., Middleton F.A., Bialosuknia S., Zhang-James Y., Liu L., Tsuang M.T., Faraone S.V., Glatt S.J. (2012). Transcriptomic analysis of postmortem brain identifies dysregulated splicing events in novel candidate genes for schizophrenia. Schizophr. Res..

[101] Sanders A.R., Göring H.H.H., Duan J., Drigalenko E.I., Moy W., Freda J., He D., Shi J., MGS, Gejman P.V. (2013). Transcriptome study of differential expression in schizophrenia. Hum. Mol. Genet..

[102] Olde Loohuis N.F., Nadif Kasri N., Glennon J.C., van Bokhoven H., Hébert S.S., Kaplan B.B., Martens G.J., Aschrafi A. (2017). The schizophrenia risk gene MIR137 acts as a hippocampal gene network node orchestrating the expression of genes relevant to nervous system development and function. Prog. Neuropsychopharmacol. Biol. Psychiatry.

[103] Collins A.L., Kim Y., Bloom R.J., Kelada S.N., Sethupathy P., Sullivan P.F. (2014). Transcriptional targets of the schizophrenia risk gene MIR137. Transl. Psychiatry.

[104] Geaghan M.P., Atkins J.R., Brichta A.M., Tooney P.A., Scott R.J., Carr V.J., Cairns M.J. (2019). Alteration of miRNA-mRNA interactions in lymphocytes of individuals with schizophrenia. J. Psychiatr. Res..

[105] Perkins D.O., Olde Loohuis L., Barbee J., Ford J., Jeffries C.D., Addington J., Bearden C.E., Cadenhead K.S., Cannon T.D., Cornblatt B.A. (2020). Polygenic Risk Score Contribution to Psychosis Prediction in a Target Population of Persons at Clinical High Risk. Am. J. Psychiatry.

[106] Moreau M.P., Bruse S.E., David-Rus R., Buyske S., Brzustowicz L.M. (2011). Altered MicroRNA Expression Profiles in Postmortem Brain Samples from Individuals with Schizophrenia and Bipolar Disorder. Biol. Psychiatry.

[107] Rodrigues-Amorim D., Rivera-Baltanás T., Vallejo-Curto M.d.C., Rodriguez-Jamardo C., de las Heras E., Barreiro-Villar C., Blanco-Formoso M., Fernández-Palleiro P., Álvarez-Ariza M., López M. (2019). Proteomics in Schizophrenia: A Gateway to Discover Potential Biomarkers of Psychoneuroimmune Pathways. Front. Psychiatry.

[108] Ramsey J.M., Schwarz E., Guest P.C., van Beveren N.J.M., Leweke F.M., Rothermundt M., Bogerts B., Steiner J., Bahn S. (2013). Distinct Molecular Phenotypes in Male and Female Schizophrenia Patients. PLoS ONE.

[109] Tomasik J., Smits S.L., Leweke F.M., Eljasz P., Pas S., Kahn R.S., Osterhaus A.D.M.E., Bahn S., de Witte L.D. (2018). Virus discovery analyses on post-mortem brain tissue and cerebrospinal fluid of schizophrenia patients. Schizophr. Res..

[110] Davalieva K., Maleva Kostovska I., Dwork A.J. (2016). Proteomics Research in Schizophrenia. Front. Cell. Neurosci..

[111] Goldman S.A., Kuypers N.J. (2015). How to make an oligodendrocyte. Development.

[112] Cerneckis J., Cai H., Shi Y. (2024). Induced pluripotent stem cells (iPSCs): Molecular mechanisms of induction and applications. Signal Transduct. Target. Ther..

